# HIF-1α at the intersection of hypoxia, ferroptosis-associated stress, and cell death crosstalk in osteomyelitis

**DOI:** 10.3389/fcell.2026.1672284

**Published:** 2026-01-30

**Authors:** Jinglin Li, Fuyin Yang, Xuan Deng, Yang Yu, Xianpeng Huang, Xuxu Yang, Lidan Yang, Tao Zhang, Huazhang Xiong

**Affiliations:** 1 Department of Orthopedics, Affiliated Hospital of Zunyi Medical University, Zunyi, China; 2 Joint Orthopaedic Research Center of Zunyi Medical University, University of Rochester Medical Center, Zunyi, China; 3 Key Laboratory of Cell Engineering of Guizhou Province, Affiliated Hospital of Zunyi Medical University, Zunyi, China

**Keywords:** apoptosis, ferroptosis, hypoxia-inducible factor-1α, inflammation, osteoimmunology, osteomyelitis, pyroptosis, regulated cell death

## Abstract

Osteomyelitis is a severe inflammatory disease of bone tissue primarily caused by bacterial infections, most commonly *Staphylococcus aureus*. Its complex pathophysiology creates a unique hypoxic and inflamed microenvironment, which leads to the significant upregulation of the key transcriptional regulator, hypoxia-inducible factor-1α (HIF-1α). HIF-1α plays a pivotal role in disease progression, partly by orchestrating various forms of regulated cell death (RCD). The dysregulation of these RCD pathways, including apoptosis, pyroptosis, and particularly the emerging role of ferroptosis, is critically involved in shaping the fate of bone and immune cells, influencing the inflammatory response, and ultimately driving bone destruction. This review aims to comprehensively explore the regulatory mechanisms of HIF-1α on these RCD modalities, especially ferroptosis, and the intricate crosstalk among them. Moreover, we highlight emerging therapeutic strategies targeting the HIF-1α-RCD axis, offering novel insights into the pathogenesis and potential treatment avenues for this refractory orthopedic inflammatory condition.

## 1 Introduction

Osteomyelitis is a severe infectious disease of bone tissue characterized by progressive inflammatory destruction and new bone formation. A wide range of microorganisms can cause osteomyelitis, among which *Staphylococcus aureus* accounts for approximately 60% of all cases. The infection can be acquired through various routes, including hematogenous spread (common in children), contiguous spread from adjacent foci, or direct contamination following trauma or surgery (more frequent in adults). Comorbidities such as diabetes and vascular insufficiency are key predisposing factors for osteomyelitis (Granata et al., 2022; Rosenberg and Khurana, 2016). The pathogenesis of osteomyelitis is a complex, multi-step process involving bacterial colonization in bone tissue, activation of the host immune response, release of inflammatory mediators, and disruption of bone homeostasis, ultimately leading to pathological changes such as bone resorption, sequestrum formation, and sinus tract development (Chen H. et al., 2022; Masters et al., 2022). Clinical management of osteomyelitis remains highly challenging due to difficulties in eradicating infection, high recurrence rates, the need for prolonged antibiotic therapy, and multiple surgical debridements, all of which severely impact patients’ quality of life and impose a substantial healthcare burden (Wang X. et al., 2023). The lesion microenvironment of osteomyelitis exhibits distinct features, including local hypoxia, acidosis, nutrient deprivation, and elevated concentrations of inflammatory cytokines and chemokines (Granata et al., 2022). This unique microenvironment arises from the synergistic effects of bacterial invasion, massive immune cell infiltration, and vascular damage. Bacteria and their products activate both innate immune cells (e.g., neutrophils and macrophages) and adaptive immune cells, resulting in the release of pro-inflammatory mediators such as tumor necrosis factor-α (TNF-α), interleukin-1β (IL-1β), and IL-6 (Li J. et al., 2025; Oliveira et al., 2020). These inflammatory factors not only directly damage bone tissue but also exacerbate hypoxia and acidosis locally. The concept of osteoimmunology highlights the intricate interactions among invading pathogens, immune cells, and skeletal cells (osteoblasts and osteoclasts) in driving the development and progression of osteomyelitis (Collet et al., 2025). Recent studies further confirm this crosstalk, indicating that in the osteomyelitis microenvironment, macrophage pyroptosis releases large amounts of inflammatory mediators (such as IL-1β). These mediators not only exacerbate the inflammatory storm but also directly disrupt the balance between osteoblasts and osteoclasts, thereby linking cell death modalities directly to bone destruction (Feng et al., 2025). The microenvironment is not merely a passive site of inflammation, but an active driver of cellular stress responses. Inflammation and immune activation triggered by bacterial infection, along with vascular injury and increased oxygen consumption, collectively contribute to local hypoxia (Veis and Cassat, 2021; Zhang et al., 2022a), a key condition for the stabilization of hypoxia-inducible factor 1-alpha (HIF-1α). Moreover, hypoxia and inflammation serve as cellular stress signals that can directly induce regulated cell death (RCD) (Chen W. et al., 2022; Lee et al., 2023). Therefore, the microenvironment in osteomyelitis actively shapes both HIF-1α signaling and cell death pathways, which are not only consequences but also integral drivers of disease progression.

**FIGURE 1:**
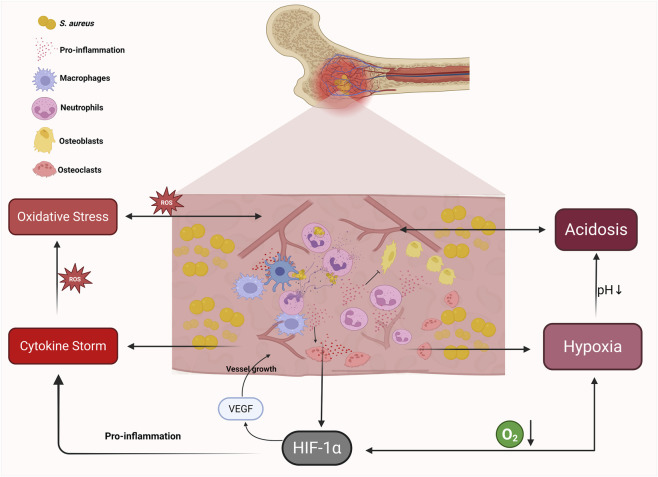
Hypoxic immune microenvironment and pathological features of osteomyelitis induced by *Staphylococcus aureus*. During *S. aureus*-induced osteomyelitis, bacterial colonization around the lesion leads to the formation of a hypoxic immune microenvironment. Macrophages secrete pro-inflammatory cytokines, while neutrophils release neutrophil extracellular traps (NETs) to eliminate invading pathogens. However, the persistent release of inflammatory mediators promotes osteoclast activation and inhibits osteoblast function, exacerbating bone destruction at the infection site. The interplay of hypoxia, reactive oxygen species (ROS)-induced oxidative stress, and inflammatory cytokines further activates hypoxia-inducible factor-1 alpha (HIF-1α), aggravating immune dysregulation and triggering regulated cell death pathways. The lesion is characterized by four key pathological features: hypoxia, acidosis, oxidative stress, and cytokine storm. HIF-1α plays a central role in orchestrating these responses, partly by enhancing angiogenic VEGF expression and amplifying the inflammatory cascade.

Regulated cell death (RCD) refers to a genetically controlled process of active cellular self-destruction, essential for maintaining tissue homeostasis and eliminating damaged or infected cells. The major forms of RCD include apoptosis, pyroptosis, ferroptosis, and necroptosis (Yuan and Ofengeim, 2024), all of which play complex roles in infection and inflammatory diseases (Lee et al., 2023). In osteomyelitis, dysregulation of RCD in various cell types—such as osteoblasts, osteoclasts, and immune cells—may contribute to bone destruction, persistent inflammation, and chronic infection. Traditionally, studies of cell death in osteomyelitis have mainly focused on apoptosis (Wang Y. et al., 2023). However, emerging non-apoptotic forms of RCD, such as ferroptosis and pyroptosis, are garnering increasing attention due to their distinctive molecular mechanisms and strong pro-inflammatory effects (Zhou SR. et al., 2025; Gao L. et al., 2025; Zhu et al., 2019). Activation of these inflammatory RCD pathways may lead to the release of cytokines and damage-associated molecular patterns (DAMPs), further amplifying inflammation and tissue injury. HIF-1α, the oxygen-sensitive subunit of the HIF-1 transcriptional complex, is stabilized under hypoxic conditions and triggers a cascade of cellular responses (Cowman and Koh, 2022). It serves as a central transcriptional regulator in response to hypoxia and inflammatory stimuli (Koh and Powis, 2012). In the hypoxic and inflamed microenvironment of osteomyelitis lesions, HIF-1α is markedly activated and upregulated (Zhang et al., 2022a; Cao et al., 2024). By regulating the expression of a wide range of target genes, HIF-1α participates in angiogenesis, energy metabolism, cell survival and death, and the inflammatory response (Chen W. et al., 2022; Zhou J. et al., 2025). It plays a critical yet complex role in the pathophysiology of osteomyelitis, influencing disease outcomes through its regulation of various RCD pathways. This review aims to systematically elucidate the molecular mechanisms by which HIF-1α regulates apoptosis, pyroptosis, ferroptosis, and other forms of RCD in osteomyelitis. We further explore the crosstalk among different RCD pathways and their integrated effects on disease progression. Based on these insights, we discuss the feasibility and prospects of targeting HIF-1α and specific RCD pathways as potential therapeutic strategies for osteomyelitis, with the goal of providing references for future basic research and clinical translation (Figure 1).

## 2 HIF-1α: a central regulator in the inflammatory microenvironment of osteomyelitis

### 2.1 Molecular biology of HIF-1α: structure, regulation, and transcriptional activity

**FIGURE 2:**
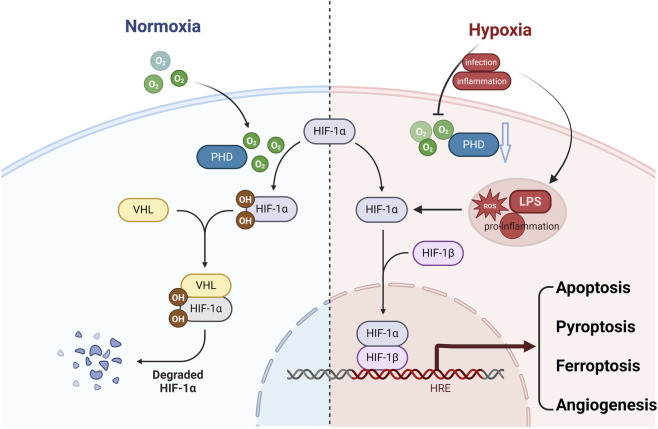
Oxygen-dependent regulation of HIF-1α and its downstream effects. Under normoxic conditions (Normoxia), specific proline residues on HIF-1α are hydroxylated by prolyl hydroxylase domain proteins (PHDs). The hydroxylated HIF-1α is then recognized by the VHL (von Hippel-Lindau) E3 ubiquitin ligase complex, leading to its ubiquitination and rapid proteasomal degradation (Degraded HIF-1α). In contrast, under hypoxic conditions (Hypoxia)—such as those induced by infection and inflammation—the activity of PHDs is suppressed, preventing HIF-1α hydroxylation and degradation. As a result, HIF-1α accumulates in the cytoplasm, translocates into the nucleus, heterodimerizes with HIF-1β, and binds to hypoxia response elements (HREs) in the promoters or enhancers of target genes. This primarily activates adaptive transcriptional programs involved in promoting angiogenesis and metabolic reprogramming. Concurrently, as discussed in this review, HIF-1α also indirectly modulates various regulated cell death (RCD) pathways—including apoptosis, pyroptosis, and ferroptosis—by regulating specific targets (e.g., NLRP3 or SLC7A11) in a context-dependent manner.

Hypoxia-inducible factor 1 (HIF-1) is a heterodimeric transcription factor composed of an oxygen-regulated α subunit (HIF-1α) and a constitutively expressed β subunit (HIF-1β, also known as ARNT) (Chen W. et al., 2022; Cowman and Koh, 2022). While HIF-1β is stable and constantly present in cells, the stability of HIF-1α is tightly controlled by intracellular oxygen levels. Under normoxic conditions, specific proline residues on HIF-1α are hydroxylated by prolyl hydroxylase domain enzymes (PHDs), a process requiring molecular oxygen (O_2_), ferrous iron (Fe^2+^), and α-ketoglutarate as cofactors (Heyman et al., 2011). The hydroxylated HIF-1α is recognized by the von Hippel-Lindau (VHL) E3 ubiquitin ligase complex, leading to its ubiquitination and subsequent rapid degradation via the proteasome pathway, thus maintaining extremely low levels of HIF-1α under normal oxygen tension (Maxwell et al., 1999; Jaakkola et al., 2001). In contrast, under hypoxic conditions, PHD activity is inhibited, preventing hydroxylation and degradation of HIF-1α. As a result, HIF-1α accumulates in the cytoplasm, translocates into the nucleus, and forms a heterodimer with HIF-1β. This HIF-1 complex binds to hypoxia-responsive elements (HREs) in the promoters or enhancers of target genes, initiating transcriptional activation (Heyman et al., 2011). HIF-1α plays a pivotal role in cellular adaptive responses to hypoxia, promoting the expression of genes involved in angiogenesis—such as vascular endothelial growth factor (VEGF), platelet-derived growth factor-B (PDGF-B), placental growth factor, stromal-derived factor-1 (SDF-1) and its receptor, angiopoietin-2—as well as various hypoxia-regulated gene products and glycolytic enzymes (Campochiaro, 2013; Viola et al., 2019). Beyond hypoxia, several non-hypoxic stimuli can also induce the accumulation and activation of HIF-1α. These include reactive oxygen species (ROS), inflammatory cytokines (e.g., IL-1β can upregulate HIF-1α expression in chondrocytes (Zeng et al., 2022), and bacterial components such as lipopolysaccharide (LPS), which can enhance HIF-1α transcription in pulpitis via the NF-κB and MAPK signaling pathways (Shao et al., 2025). These non-hypoxic activation mechanisms are particularly relevant in inflammatory conditions such as osteomyelitis (Figure 2).

### 2.2 HIF-1α in bone homeostasis

#### 2.2.1 Osteoblasts

**FIGURE 3:**
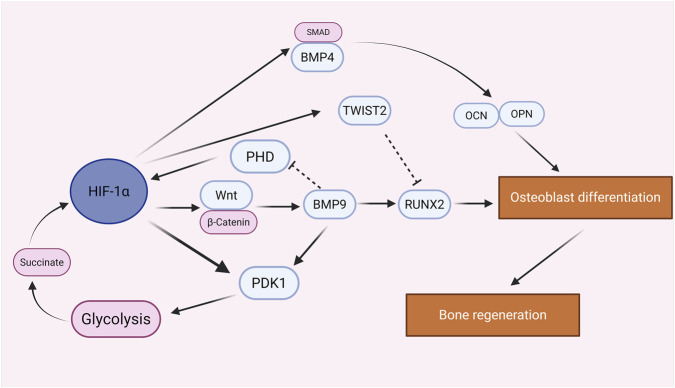
Regulation of osteoblasts by HIF-1α in bone homeostasis.

HIF-1α plays a multifaceted role in maintaining skeletal homeostasis. It is involved in regulating the osteogenic differentiation of bone marrow-derived mesenchymal stem cells (BMSCs) (Chen W. et al., 2022) and influences glucose metabolism in osteoblasts (Qiu et al., 2024). Studies have shown that overexpression of HIF-1α counteracts hypoxia-induced apoptosis in osteoblasts, thereby enhancing cell viability (XU et al., 2015), suggesting a protective role for HIF-1α in osteoblasts under hypoxic conditions. The role of HIF-1α in osteoblast differentiation is complex and highly stage-dependent. On one hand, overexpression of HIF-1α has been reported to suppress osteogenic differentiation. Through RNA-seq analysis, Lee et al. demonstrated that HIF-1α overexpression significantly downregulates key osteogenic markers such as Runx2 and osteocalcin (Ocn). Mechanistically, HIF-1α was found to upregulate the transcriptional repressor Twist2, which interferes with RUNX2 activity, thereby inhibiting osteogenesis (Lee et al., 2024). On the other hand, conditional knockout of the VHL gene in osteoblasts, which leads to HIF-1α accumulation, promotes long bone formation and vascularization, suggesting that during developmental stages, HIF-1α enhances bone formation by stimulating angiogenesis (Wang et al., 2007). These seemingly contradictory findings indicate a critical dual regulatory function of HIF-1α in osteoblasts, where the net effect thedepends on the mechanistic context and cellular stage: On one hand, HIF-1α can inhibit osteoblast maturation through direct transcriptional repression (e.g., upregulating Twist2 to interfere with RUNX2 activity). On the other hand, in developmental or angiogenesis-driven contexts (as seen in the VHL knockout), HIF-1α′s primary contribution is indirect promotion by stimulating angiogenesis (e.g., via the HIF-1α/VEGF axis), which provides the essential vascular supply for bone formation. Therefore, the ultimate role of HIF-1α is the result of a complex balance between its direct cell-autonomous inhibitory effects and its indirect, microenvironment-driven pro-osteogenic effects. In addition, HIF-1α modulates osteogenesis by regulating osteogenic signaling pathways. Under hypoxic conditions, HIF-1α expression is elevated and activates the BMP4/SMAD pathway, enhancing the expression of osteogenic markers such as OCN and OPN, and thereby promoting osteogenic differentiation of BMSCs (Chen et al., 2024a). BMP9, a promising growth factor in bone tissue engineering, has also been implicated in HIF-1α-mediated osteogenic regulation. Zhang et al. demonstrated that inhibition of HIF-1α suppresses Wnt/β-catenin signaling, thereby reducing BMP9-induced osteogenic differentiation (Zhang J. et al., 2023). Hypoxia regulates BMP signaling components and targets without altering BMP9 levels; moreover, conditioned medium derived under hypoxic conditions promotes cell migration and osteogenic differentiation. BMP9 enhances HIF-1α expression, and knockdown of HIF-1α attenuates BMP9-induced osteoblast differentiation via regulation of its downstream target Runx2 (Paz et al., 2023; Li et al., 2022). These findings open new avenues for modulating the BMP9–HIF-1α axis in cell-based therapies aimed at promoting bone regeneration. Enhanced glycolysis is a hallmark of differentiating osteoblasts. By upregulating HIF-1α in BMSCs, glycolytic activity is increased, which in turn promotes osteoblast differentiation under high-glucose conditions (Zhang S. et al., 2025; Lee et al., 2020), thereby accelerating bone healing. A study by Muhammad Subhan Amir and colleagues further confirmed the critical role of HIF-1α in BMP9-mediated osteoblast differentiation, a process dependent on the induction of pyruvate dehydrogenase kinase 1 (PDK1). Specifically, BMP9 increases HIF-1α expression by inhibiting PHD activity in osteoblasts. Elevated HIF-1α levels are essential for upregulating PDK1, which in turn serves as a key metabolic regulator required for BMP9-driven osteogenic differentiation (Amir et al., 2022) (Figure 3).

#### 2.2.2 Osteoclasts

**FIGURE 4:**
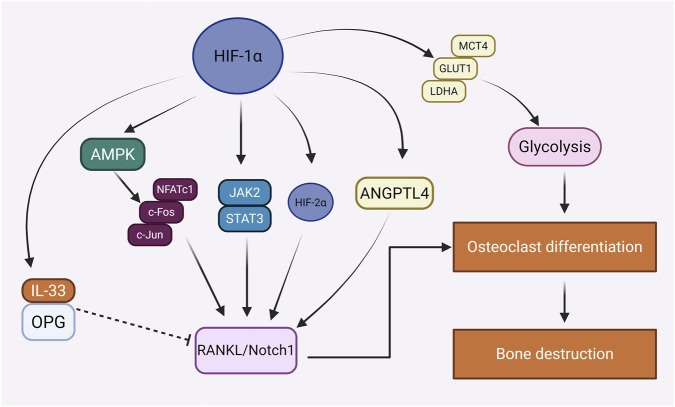
Regulation of osteoclasts by HIF-1α in bone homeostasis.

The role of HIF-1α in osteoclasts is highly complex and dualistic. On the one hand, activation of HIF-1α in osteoblasts has been shown to suppress osteoclastogenesis by upregulating osteoprotegerin (OPG) and interleukin-33 (IL-33), thereby inhibiting the RANKL/Notch1 signaling pathway (Kang et al., 2017). On the other hand, HIF-1α can also enhance RANKL expression via the JAK2/STAT3 signaling pathway, directly promoting RANKL-mediated osteoclast differentiation. HIF-1α exerts both direct and indirect effects on osteoclastogenesis. Directly, it has been shown to enhance osteoclast differentiation (Zhang C. et al., 2023; Miyauchi et al., 2013; Wang D. et al., 2022). For instance, stabilization of HIF-1α in RAW264.7 cells using an iron chelator (L-valine hydroxamate) enhances RANKL-induced osteoclast formation, accompanied by activation of the MAPK signaling pathway and increased expression of osteoclast-specific transcription factors such as NFATc1, c-Fos, and c-Jun (Wang D. et al., 2022). Angiopoietin-like protein 4 (ANGPTL4), a hypoxia-induced adipokine, also promotes osteoclast activity and bone resorption *in vitro*. Xin Qi et al. reported that suppression of HIF-1α expression reduces ANGPTL4 levels, indicating that HIF-1α promotes osteoclastogenesis through ANGPTL4 (Qi et al., 2023). Additionally, inhibition of HIF-1α reduces the expression of glycolysis-related proteins such as GLUT1, LDHA, and MCT4, thereby impairing osteoclast differentiation and resorptive activity (Nishioku et al., 2025). It has also been proposed that the anti-inflammatory agent Iguratimod may modulate the AMPK/HIF-1α signaling axis, thereby suppressing inflammatory cytokine release and altering osteoclast differentiation (Ying et al., 2025). HIF-1α also indirectly regulates osteoclastogenesis by modulating the expression of RANKL and OPG in osteoblasts, thus affecting the RANKL/OPG ratio (Chen W. et al., 2022). For example, Lee et al. demonstrated that overexpression of HIF-1α in osteoblasts significantly upregulated RANKL expression without affecting OPG levels, thereby increasing the RANKL/OPG ratio and enhancing osteoclastogenic potential. Interestingly, this upregulation of RANKL was not a direct effect of HIF-1α itself, but rather mediated through induction of its downstream target HIF-2α. Specifically, HIF-1α directly binds to the *Hif2a* promoter, leading to HIF-2α upregulation, which in turn promotes RANKL expression (Lee et al., 2024). HIF-1α may also inhibit osteoclast activity through other mechanisms. Following HIF-1α-induced osteocyte apoptosis, surviving osteocytes release RANKL and VEGF; concurrently, HIF-1α upregulates anti-resorptive factors such as OPG and IL-33, thereby suppressing osteoclastic gene expression via the RANKL/Notch1 pathway (Kang et al., 2017; Hulley et al., 2017). Moreover, catharanthine tartrate (CAT), a compound that destabilizes HIF-1α, has been shown to effectively inhibit osteoclast activity and ameliorate RANKL-induced bone loss. Mechanistically, CAT promotes ubiquitination and proteasomal degradation of HIF-1α, thereby attenuating osteoclast differentiation and bone resorption (Cai L. et al., 2025). Taken together, HIF-1α exerts bidirectional effects on osteoclasts. In certain high-energy-demanding and inflammatory contexts, HIF-1α acts synergistically with RANKL to promote osteoclast differentiation and bone resorption. In contrast, under physiological conditions, HIF-1α in mature osteoblasts may suppress osteoclast activity by enhancing OPG expression or engaging in negative feedback signaling. These findings underscore the importance of a finely tuned balance between HIF-1α and the RANKL/OPG axis in maintaining bone homeostasis (Figure 4).

#### 2.2.3 HIF-1α promotes osteogenic angiogenesis

**FIGURE 5:**
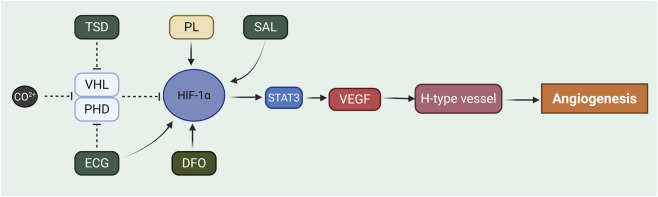
Regulation of Angiogenesis by HIF-1α in bone homeostasis. This diagram illustrates the signaling network centered on HIF-1α as a pivotal regulator of angiogenic-osteogenic coupling. Under hypoxia or pharmacological intervention, HIF-1α protein is stabilized. For example, CO^2+^ (Cobalt ions) and DFO (Deferoxamine, an iron chelator) can stabilize HIF-1α by inhibiting the activity of prolyl hydroxylases (PHDs) and the VHL (von Hippel-Lindau) protein, thereby preventing HIF-1α degradation. Additionally, various natural compounds and biological agents, such as TSD (Tao-Hong-Si-Wu decoction), SAL (Salidroside), ECG (Epicatechin gallate), and PL (Platelet lysate), have been shown to activate or upregulate the HIF-1α signaling pathway through different mechanisms. Stabilized HIF-1α, in concert with pathways like STAT3, then promotes the transcription and expression of its key downstream target, VEGF (Vascular Endothelial Growth Factor). The upregulation of VEGF is a critical driver for the formation of H-type vessels (a specialized capillary subtype tightly associated with osteogenesis), ultimately leading to Angiogenesis.

HIF-1α serves as a pivotal regulator in the coupling of angiogenesis and osteogenesis. By upregulating pro-angiogenic factors such as VEGF, HIF-1α promotes the formation of new blood vessels, thereby supplying essential nutrients and oxygen for bone repair and regeneration (Zhang et al., 2022a). For instance, cobalt ions (Co^2+^) can inhibit the degradation of HIF-1α by inactivating prolyl hydroxylases (PHDs) and preventing the interaction between HIF-1α and the VHL complex, which subsequently enhances VEGF expression and stimulates both osteogenesis and angiogenesis (Fan et al., 2010; Yuan et al., 2003). Similarly, deferoxamine (DFO) stabilizes and enhances HIF-1α, thereby promoting mineralization, angiogenesis, and osteogenic differentiation of bone marrow stromal cells (BMSCs) (Lang et al., 2022). PHD inhibitors reduce HIF-1α degradation and modulate its downstream target genes, contributing to increased angiogenesis, proliferation, migration, and osteogenic differentiation of BMSCs, while also inhibiting osteoblast apoptosis (Zhang et al., 2018; Wang X. et al., 2022). The HIF-1α/VEGF axis plays a central role in “angiogenic-osteogenic coupling,” a process critical for fracture healing and bone defect repair. However, under pathological conditions such as osteomyelitis, aberrant angiogenesis may facilitate the persistence and dissemination of inflammation. In the bone marrow, a specialized capillary subtype known as H-type vessels—characterized by high expression of endomucin and CD31—has been shown to be tightly associated with osteogenesis and regulated by HIF-1α (Chen W. et al., 2022; Liu et al., 2023). Studies have demonstrated that in young mice, endothelial cells of H-type vessels express high levels of HIF-1α; with aging, both HIF-1α expression and H-type vessel abundance decline, accompanied by reduced bone mass (Kusumbe et al., 2014). Endothelial cell-specific deletion of HIF-1α in mice leads to decreased osteoprogenitor populations and the development of osteoporosis. Conversely, overexpression of HIF-1α in osteoblasts enhances both angiogenesis and bone formation (Wan et al., 2010). In pathological bone metabolic conditions, stabilizing HIF-1α has emerged as a promising therapeutic strategy for bone regeneration. For example, PHD inhibitors such as DFO can prevent HIF-1α degradation, thereby enhancing H-type vessel formation and bone repair in ischemic or bone defect models (Liu et al., 2023; Qin et al., 2022). VEGF plays a vital role in vascular formation and remodeling, and HIF-1α can directly induce VEGF transcription and translation in hypoxic or ischemic cells, significantly promoting H-type vessel formation in the metaphysis (Zhang J. et al., 2020). Additionally, various herbal compounds have been shown to modulate the HIF-1α/VEGF signaling pathway within the bone microenvironment to enhance angiogenic-osteogenic coupling. For example, salidroside (SAL) has been shown to directly upregulate HIF-1α expression and increase its transcriptional activity, consequently upregulating VEGF expression at both the mRNA and protein levels, thereby promoting angiogenesis-osteogenesis coupling (Guo et al., 2020). Tao-Hong-Si-Wu decoction (TSD) has demonstrated a similar mechanism in models of avascular necrosis of the femoral head, where it significantly promoted the expression of HIF-1α and VEGF (Tang Z. et al., 2022). TSD may also induce VEGF production via the PI3K/Akt-eNOS signaling pathway, which is known to act upstream of HIF-1α. Furthermore, epicatechin gallate (ECG), when applied to a co-culture system of osteoblasts and endothelial cells, was found to enhance microvessel formation by significantly increasing the levels of pro-angiogenic factors, including VEGF and PDGF-BB. The authors of that study concluded that this effect is “probably via HIF signaling” (Zhang L. et al., 2023). These studies collectively suggest the potential of herbal compounds targeting the HIF-1α/VEGF axis to accelerate fracture healing. Moreover, platelet lysate (PL) has been reported to enhance VEGF secretion by stimulating STAT3 phosphorylation and robust induction, nuclear translocation, and DNA-binding activity of HIF-1α in osteoblasts, promoting both angiogenesis and bone formation (Nguyen et al., 2020). Some studies on bone marrow-derived mesenchymal stem cell-derived exosomes (BMMSC-Exos) have also shown their ability to activate HIF-1α/VEGF and BMP-2/Smad1/RUNX2 signaling pathways, facilitating osteogenesis and angiogenesis, and playing a key role in the treatment of nonunion fractures (Zhang L. et al., 2020). As a key driver of angiogenesis and bone remodeling, HIF-1α plays a critical role in bone healing (Figure 5).

### 2.3 The role of HIF-1α in immune cells: regulation of inflammatory responses

#### 2.3.1 Macrophages

HIF-1α plays a pivotal role in regulating macrophage function during bone infection and inflammation. During the inflammatory process, macrophage metabolism undergoes a shift from oxidative phosphorylation to glycolysis, with HIF-1α emerging as a central regulator. Upon stimulation by *S. aureus* protein A in osteomyelitis, macrophages exhibit increased expression of HIF-1α, which subsequently activates glycolysis and promotes polarization toward the pro-inflammatory M1 phenotype (Li J. et al., 2025; Zhu et al., 2024). In addition, oxygen consumption by bacterial metabolism can lead to local hypoxia, inhibiting prolyl hydroxylase domain (PHD) activity and allowing HIF-1α to accumulate and translocate to the nucleus (Zenk et al., 2021). HIF-1α drives metabolic reprogramming in macrophages by upregulating glucose transporters and glycolytic enzymes, thereby providing a rapid energy supply that enhances M1 polarization and exacerbates inflammation (Han et al., 2025). In models of *Mycobacterium* infection, macrophages with elevated HIF-1α expression demonstrate enhanced phagocytic and bactericidal activity (Zenk et al., 2021). Pharmacological stabilization of HIF-1α has been shown to induce expression of the vitamin D receptor and antimicrobial peptide hBD-2, while simultaneously reducing the release of TNF-α and IL-10, thereby significantly inhibiting bacterial proliferation (Nickel et al., 2012). Collectively, these findings indicate that HIF-1α enhances the antimicrobial and immunoregulatory capacity of macrophages. In the context of bone infection, HIF-1α-mediated inflammatory mediators and metabolic byproducts exert multiple effects on bone remodeling. On one hand, M1 macrophage-derived cytokines such as IL-6 and TNF-α stimulate osteoclast differentiation and activation, promoting bone resorption (Meng et al., 2021). On the other hand, HIF-1α-enhanced M1 polarization can indirectly inhibit osteoblast differentiation via the BMP4 signaling pathway, impairing bone formation (Zhu et al., 2024). HIF-1α also induces the expression of pyruvate dehydrogenase kinase 1 (PDK1), initiating a glucose metabolic reprogramming known as the “Pasteur effect” (Webster, 2003). HIF-1α is thus regarded as a master regulator linking enhanced glycolysis in M1 macrophages to inflammatory responses (McGettrick and O’Neill, 2020). Studies have demonstrated that activation of glycolysis via the HIF-1α/PDK1 axis significantly enhances macrophage migration and M1 polarization during the inflammatory phase (Lin et al., 2024). Notably, Kelvin Ka-lok Wu and colleagues reported that MDM2 promotes degradation of SPSB2, leading to iNOS expression and NO production, which further increases HIF-1α activity and its dependency for glycolytic metabolism and inflammatory cytokine production in M1 macrophages (Wu et al., 2024). The Wnt/β-catenin signaling pathway also interacts with HIF-1α to promote glycolytic metabolism and inflammatory activation in macrophages. For instance, Bibo Zhu and colleagues discovered that Wnt signaling in tissue-resident alveolar macrophages (AMs) leads to the formation of an “unconventional” β-catenin–HIF-1α complex, which promotes glycolysis-dependent inflammation (Zhu et al., 2021). Inhibiting the Wnt/β-catenin pathway can significantly downregulate HIF-1α and suppress the polarization of macrophages from the anti-inflammatory M2 phenotype to the pro-inflammatory M1 phenotype (Chen et al., 2024b). Whether similar signaling mechanisms exist in macrophages within osteomyelitic lesions remains an open question warranting further investigation.

#### 2.3.2 Neutrophils

HIF-1α also enhances the bactericidal function of neutrophils. For instance, in pulpitis, it promotes the release of neutrophil extracellular traps (NETs), thereby facilitating neutrophil recruitment and enhancing their antimicrobial capacity (Shao et al., 2025). Under hypoxic conditions, HIF-1α is stabilized and translocated into the nucleus of neutrophils, leading to increased activation of NF-κB, a key transcription factor regulating neutrophil survival and cytokine production. Elevated HIF-1α expression in neutrophils has been shown to enhance phagocytic capacity and significantly prolong neutrophil survival (Dölling et al., 2022). *In vivo* studies demonstrate that hypoxia-induced upregulation of HIF-1α in neutrophils extends their retention time at infection sites, thereby enhancing the immune response (Ye et al., 2025; Lin and Simon, 2016). Neutrophils rely heavily on anaerobic glycolysis for energy. Upon HIF-1α activation, key glycolytic enzymes are upregulated, boosting cellular energy production (Thind et al., 2023). Lactate, a major byproduct of neutrophil glycolysis, serves as a clinical marker for sepsis (Liu et al., 2019). HIF-1α-mediated lactate production and neutrophil mobilization are critical during acute inflammation. Disruption of a single HIF-1α allele impairs lactate generation and release from activated neutrophils and reduces their mobilization into circulation (Khatib-Massalha et al., 2020). Recent studies show that the HIF-1α-dependent glycolytic pathway significantly enhances NET formation under hypoxic conditions, enabling effective pathogen capture and elimination via NETosis (Ye et al., 2025). NETs also promote HIF-1α expression through TLR4 activation. HIF-1α, in turn, upregulates MMP-9 and IL-1β, promoting inflammation and angiogenesis (Zeng et al., 2023). By increasing HIF-1α stability and activity in hypoxic tissues, NETs amplify the inflammatory response and vascular growth, ultimately exacerbating tissue damage and delaying normal healing (Zhang H. et al., 2023). Additionally, HIF-1α regulates neutrophil chemotaxis, adhesion, and phagocytosis (Cramer et al., 2003). Stabilized HIF-1α enhances β2 integrin expression, promoting adhesion and migration, while also boosting phagocytic activity and oxidative burst, thereby improving bacterial clearance (Willson et al., 2022). A study by Huiying Lu et al. demonstrated that increased HIF-1α expression and glycolytic activity in neutrophils suppresses their migration, apoptosis, and the release of ROS, MPO, antimicrobial peptides, and IL-8, effectively preventing excessive neutrophil activation and inflammatory damage during infection (Lu et al., 2021). Under hypoxic conditions where HIF-1α is stabilized, both human and murine neutrophils show elevated expression of MIF and IL-23—key upstream cytokines of type 3 immunity. This promotes type 3 immune inflammation and supports chondrogenesis through STAT3 signaling, a process closely linked to HIF-1α expression (Nakamura et al., 2024). Collectively, HIF-1α enables neutrophils to function more effectively in hypoxic and inflammatory environments through metabolic reprogramming and proinflammatory signaling (Kaplan, 2022).

#### 2.3.3 Other immune cells

HIF-1α also plays a regulatory role in various other immune cells, including regulatory T cells (Tregs), although most current studies have focused on autoimmune diseases (Chen et al., 2024c). Nevertheless, these findings suggest a potentially broad role for HIF-1α in the immunoregulation of osteomyelitis. T helper 17 (Th17) cells and Tregs are key modulators in maintaining the balance of inflammatory responses. Bacterial infections increase HIF-1α expression, along with elevated frequencies of both Th17 and Treg cells (Chen L. et al., 2023). A study by Marie Groneberg et al. revealed that in male mice, hepatic HIF-1α can modulate Th17 cells via IL-6 signaling. In the absence of HIF-1α in hepatocytes, abscess formation was significantly reduced, suggesting that HIF-1α contributes to the immunopathogenesis of abscess development (Groneberg et al., 2022). Inflammatory tissues are often hypoxic. In an inflammation model, Lu Yu and colleagues demonstrated that inhibition of HIF-1α increased the proportion of Treg cells, restored the balance of CD4^+^ T cell subsets, and decreased pro-inflammatory cytokine production. These findings suggest that HIF-1α may be a potential therapeutic target for ameliorating excessive inflammation (Yu et al., 2024). Mechanistically, HIF-1α promotes Th17 differentiation by activating the transcription factor RORγt and inhibits Treg differentiation by inducing the degradation of Foxp3, thereby regulating the Th17/Treg balance (Dang et al., 2011). While Th17 cells promote inflammation by secreting pro-inflammatory cytokines such as IL-17, IL-23, and IL-22, Tregs exert anti-inflammatory effects by releasing TGF-β and IL-10 and suppressing the functions of other immune cells (Lee, 2018). In the context of bone infection and inflammation, the HIF-1α–Th17/IL-17 axis not only enhances antibacterial immunity but also promotes osteoclast activation and bone resorption via IL-17. In contrast, HIF-1α deficiency favors Treg expansion and suppresses osteoclastic activity. Overall, the Th17/Treg imbalance regulated by HIF-1α represents a critical intersection between immune responses and bone remodeling in bone-related inflammation. Studies on dendritic cells (DCs) have similarly concentrated on autoimmune conditions. A study by Liliana M. Sanmarco’s group found that lactate produced by activated DCs and other immune cells can suppress ROS generation by inducing NDUFA4L2 expression via a HIF-1α–dependent pathway. This, in turn, downregulates the XBP1-driven transcriptional program in DCs, thereby modulating the differentiation of pathogenic autoimmune T cells. These findings highlight the importance of the lactate–HIF-1α–NDUFA4L2 axis as a novel immunoregulatory mechanism in DCs and suggest its potential as a therapeutic target for T cell–mediated autoimmune diseases (Sanmarco et al., 2023). Upon pathogen recognition, DCs activate HIF-1α–mediated aerobic glycolysis in a TLR2-dependent manner, enhancing their migratory and antimicrobial capacities (Maio et al., 2024). DC-derived cytokines, such as IL-6, together with HIF-1α signaling, promote the differentiation of naïve CD4^+^ T cells toward the Th17 lineage (Groneberg et al., 2022). Additionally, hypoxia promotes the expression of inhibitory molecules on DCs and favors a Th2-biased immune response (Xiong et al., 2016). Although research on HIF-1α in DCs remains limited, its activation can influence T cell–mediated immunity by altering cytokine profiles and migratory behavior. In the bone infection microenvironment, HIF-1α–mediated modulation of DC function may contribute to shaping the intensity and nature of the immune response (Table 1).

**TABLE 1:** HIF-1α-mediated regulation of cell death pathways in osteomyelitis-associated cells.

Cell type	Cell death pathway	HIF-1α-regulated molecular targets/mediators	Regulatory effect of HIF-1α	Impact on cell fate/function in osteomyelitis
Osteoblasts	Apoptosis	Bcl-2 family proteins, metabolic enzymes	Inhibits apoptosis, promotes cell survival	Enhances osteoblast viability, potentially preserving bone formation
	Ferroptosis	SLC7A11, GPX4	Upregulates SLC7A11/GPX4 (inhibits ferroptosis)	Suppresses ferroptosis, protects osteoblasts from oxidative damage
Macrophages	Pyroptosis	NLRP3 inflammasome, GSDMD	Upregulates NLRP3 (promotes/initiates pyroptosis)	Enhances macrophage pyroptosis, increases IL-1β release, exacerbates inflammation and bone destruction
Neutrophils	Ferroptosis	TFRC (promotes); SLC7A11/GPX4 (inhibits)	Complex/dual regulation	Promotes ferroptosis indirectly through iron uptake and inflammatory activation
	Apoptosis	Balance of pro-/anti-apoptotic proteins	Delays apoptosis	Prolongs neutrophil survival and function, potentially enhancing antibacterial capacity or inflammation

### 2.4 Upregulation and activation of HIF-1α in osteomyelitis

The hypoxic microenvironment within osteomyelitis lesions is a primary driver of HIF-1α stabilization. During the pathogenesis of osteomyelitis, HIF-1α expression and activity are significantly upregulated. Particularly in *S. aureus*-induced osteomyelitis, elevated HIF-1α levels have been observed both in infected cell models and in murine samples (Zhang et al., 2022a; Cao et al., 2024). A similar phenomenon occurs in peri-implantitis, a condition sharing pathological features with osteomyelitis, where sustained inflammation-induced hypoxia leads to increased HIF-1α expression, upregulation of VEGF, and enhanced angiogenesis, contributing to persistent inflammation (Bertoldo et al., 2024). In osteomyelitis, activated HIF-1α influences disease progression through multiple mechanisms. For instance, it promotes inflammation by upregulating transforming growth factor-β1 (TGF-β1), which in turn negatively affects osteogenesis and mineralization (Zhang et al., 2022a). In *S. aureus*-infected murine models, elevated HIF-1α expression in bone tissue is accompanied by increased serum levels of TGF-β1, IL-6, IL-1β, and C-reactive protein (Corcoran and O’Neill, 2016). Inhibition of HIF-1α significantly reduces these inflammatory markers and mitigates bone destruction. Clinically, patients with osteomyelitis exhibit markedly higher levels of HIF-1α and TGF-β1 in bone tissues or serum compared to healthy controls (Zhang et al., 2022a). Interestingly, mice with osteoblast-specific deletion of *Vhl*—resulting in constitutive HIF-1α activation—demonstrate higher baseline bone mass and reduced bone loss following infection. This protective effect appears to involve alterations in the RANKL/OPG ratio, suggesting that persistent HIF-1α activation may help preserve bone mass under certain conditions (Ford et al., 2022). Collectively, these findings underscore the pivotal role of HIF-1α signaling in osteomyelitis, serving as a key regulator of both inflammation and bone homeostasis. HIF-1α also plays a critical role in antimicrobial peptide (AMP) expression. Knockdown of HIF-1α significantly reduces human β-defensin-1 (hBD-1, DEFB1) levels (Kelly et al., 2013), while pharmacological activation of HIF-1α induces expression of LL-37 (and its murine homolog CRAMP), enhancing bactericidal activity (Fan et al., 2015). Therefore, HIF-1α not only amplifies inflammatory signaling but also boosts innate immune effector functions via AMP upregulation. In the context of bone remodeling, HIF-1α suppresses osteoblast differentiation. In osteoblasts infected with *S. aureus*, HIF-1α upregulation is associated with increased TGF-β1 expression and a concurrent reduction in osteogenic markers such as Runx2 and osteopontin (OPN), leading to diminished osteoblastic activity (Zhang et al., 2022a; Cao et al., 2024; Zhu et al., 2024). Inhibition of either HIF-1α or TGF-β1 restores osteogenic potential, indicating that HIF-1α-mediated signaling pathways hinder bone formation. Conversely, HIF-1α promotes osteoclast differentiation and function. *In vitro* studies have shown that stabilizing HIF-1α in the presence of RANKL significantly enhances osteoclastogenesis in RAW264.7 cells and upregulates genes such as cathepsin K and TRAP through the MAPK pathway, accelerating bone resorption (Liu X. et al., 2022). Moreover, HIF-1α affects osteoclast-regulatory factors secreted by osteoblasts. Transgenic mice with osteoblast-specific *Vhl* deletion maintain trabecular bone volume after infection due to a shift in the RANKL/OPG ratio (reduced RANKL and increased OPG) (Ford et al., 2022). These findings suggest that HIF-1α exerts dual regulatory effects on bone remodeling—suppressing bone formation while promoting bone resorption—ultimately exacerbating bone loss. In *S. aureus*-induced osteomyelitis, HIF-1α can directly bind to hypoxia response elements (HREs) in the *TGF-β1* mRNA promoter, inducing its expression. TGF-β1, a potent immunoregulatory cytokine, further promotes either fibrotic or reparative responses in osteomyelitis, impacting bone remodeling (Zhang et al., 2022a). Additionally, HIF-1α is a major inducer of vascular endothelial growth factor (VEGF), which drives neovascularization in hypoxic and ischemic regions (Corcoran and O’Neill, 2016). By upregulating VEGF, HIF-1α activates the VEGF/AKT/mTOR signaling cascade, promoting osteogenesis (Xu et al., 2019). Studies have also shown that enhancing HIF-1α expression in adipose-derived stem cells (ADSCs) improves both osteogenic and angiogenic capacities, highlighting HIF-1α as a key transcriptional regulator of cellular function via the HIF-1α/VEGF/AKT/mTOR pathway (Song S. et al., 2023). Thus, beyond its roles in immunity and bone metabolism, HIF-1α supports local tissue repair through VEGF-mediated angiogenesis. In summary, HIF-1α serves as a central link connecting hypoxia, immune inflammation, and bone metabolism in osteomyelitis. Infection-induced hypoxia and inflammatory stimuli upregulate HIF-1α, which then enhances immune cell metabolism and inflammatory responses, while disrupting osteoblast/osteoclast balance to aggravate bone destruction. Through its crosstalk with TGF-β1 and VEGF signaling pathways, HIF-1α contributes to both the inflammatory progression and reparative processes in osteomyelitis.

## 3 The landscape of regulated cell death in the hypoxic osteomyelitis microenvironment

### 3.1 Apoptosis: a classical pathway in bone infection

In the hypoxic osteomyelitic lesion where HIF-1α signaling is highly active—apoptosis serves as a classical non-inflammatory clearance mechanism. It is characterized by cell shrinkage, chromatin condensation, DNA fragmentation, and the formation of membrane-bound apoptotic bodies, which are subsequently cleared by macrophages without triggering a strong inflammatory response. This process can be initiated through two major pathways: the extrinsic pathway mediated by death receptors and the intrinsic pathway involving mitochondria, both ultimately converging on the activation of caspase family proteases (Newton et al., 2024; Bertheloot et al., 2021). In osteomyelitis, *S. aureus* (*S. aureus*), the predominant causative pathogen, can induce apoptosis via multiple mechanisms during infection (Chen H. et al., 2022). For instance, *S. aureus* protein A (SpA) promotes apoptosis through the induction of TNF-α and nitric oxide (NO), upregulation of pro-apoptotic factors (p53 and Bax), and downregulation of anti-apoptotic molecules such as Bcl-2. Additionally, *S. aureus* secretes nucleases (Nuc) and adenosine synthase A (AdsA) to activate caspase-3 and induce apoptotic signaling (Das et al., 2002). Another virulence factor, Staphopain B, selectively cleaves CD11b on phagocytic cells, leading to the externalization of phosphatidylserine and expression of annexin I, thereby promoting phagocyte apoptosis (Smagur et al., 2009). In the immune response to *S. aureus*, EsxA modulates cytokine production and apoptotic signaling, and in conjunction with EsxB, facilitates bacterial escape from host cells by inducing apoptosis (Korea et al., 2014). Through both apoptosis-dependent and -independent pathways, *S. aureus* impairs osteoblast activity and viability (Josse et al., 2015). Specifically, SpA interacts with osteoblasts via TNF receptor 1 (TNFR1), initiating caspase-3-dependent apoptosis and suppressing bone formation (Wen et al., 2024; Claro et al., 2011). Moreover, SpA–TNFR1 engagement activates the NF-κB and JNK pathways, promoting IL-6 expression and thereby enhancing inflammation, osteoclastic bone resorption, and osteoblast apoptosis (Kant et al., 2011; Ning et al., 2011a). Notably, genetic deletion of TNFR1 not only suppresses JNK activation but also reduces TNF-α and IL-6 secretion during infection, leading to decreased osteoblast apoptosis (Chen et al., 2016). In addition to affecting osteoblasts, *S. aureus* activates inflammasomes and recruits neutrophils to drive inflammatory responses (Kitur et al., 2016; Robinson et al., 2018). Neutrophil apoptosis is a critical step for inflammation resolution. Interestingly, a study by Jianxu Wei et al. demonstrated that potassium-doped MnO_2_ nanoparticles could reprogram calcium signaling in neutrophils, reducing intracellular ROS generation, delaying apoptosis, and sustaining neutrophil extracellular trap (NET) formation, thereby accelerating the healing of methicillin-resistant *S. aureus* (MRSA)-infected wounds (Wei et al., 2025). However, in certain contexts, such as under the activation of hypoxia-inducible factor-1α (HIF-1α), neutrophil apoptosis can be delayed. While this prolongs neutrophil survival at infection sites and may enhance bacterial clearance, it may also contribute to tissue damage due to excessive inflammation.

### 3.2 Pyroptosis: a pro-inflammatory cell death process that promotes bone destruction

Driven by the same inflammatory and hypoxic stresses that stabilize HIF-1α, pyroptosis emerges as a pro-inflammatory programmed cell death mediated by inflammatory caspases, primarily Caspase-1, Caspase-4/5 (in humans), and Caspase-11 (in mice). The hallmark of pyroptosis is the cleavage of gasdermin D (GSDMD), which releases its pore-forming N-terminal fragment (GSDMD-N). This fragment inserts into the plasma membrane to form pores, leading to ionic imbalance, cell swelling, membrane rupture, and the release of intracellular inflammatory contents, including mature IL-1β and various danger-associated molecular patterns (DAMPs) (Newton et al., 2024; McKenzie et al., 2020; Vasudevan et al., 2023). These mediators act as potent inflammatory signals that recruit additional immune cells, thereby amplifying the inflammatory response. In osteomyelitis, pyroptosis has emerged as a key mechanism driving inflammation and bone destruction. In a cell model of osteomyelitis using human bone marrow-derived mesenchymal stem cells (hBMSCs) treated with Staphylococcal protein A (SpA), levels of classic pyroptosis-associated cytokines such as IL-1β and IL-18 were significantly elevated. Knockout of Pmepa1 reversed SpA-induced cellular injury and the inhibition of osteogenic differentiation in hBMSCs by reducing IL-1β and IL-18 levels, primarily through downregulation of the p38 MAPK/NLRP3 signaling axis (Li M. et al., 2023). Studies have shown that the expression of pyroptosis-associated proteins—such as the NLRP3 inflammasome, activated Caspase-1, and GSDMD-N—is significantly elevated in infected bone tissues from osteomyelitis patients and in murine models of osteomyelitis (Zhu et al., 2019). Pyroptosis of immune cells, particularly macrophages, is a major source of IL-1β and other inflammatory cytokines, thereby directly fueling the inflammatory cascade and promoting osteolysis (Xiong et al., 2024). In pulpitis, a disease with pathological features similar to osteomyelitis, HIF-1α was identified as a positive regulator of the NLRP3 inflammasome pathway (Shao et al., 2025), suggesting that HIF-1α may also contribute to osteomyelitis progression via pyroptosis regulation. Similarly, in peri-implantitis, pyroptosis of macrophages and fibroblasts triggered by inflammatory stimuli plays a key pathogenic role (Chen L. et al., 2024). In osteoblasts, inflammasome activation during pyroptosis enhances the release of inflammatory cytokines, upregulates RANKL, and downregulates OPG, significantly increasing osteoclast activity and exacerbating bone loss (Wu et al., 2021; Roper et al., 2020). *In vitro*, pyroptotic osteoblasts exhibit diminished mineralization capacity and increased RANKL expression, thereby promoting osteoclastogenesis (Tao et al., 2020). Inhibition of pyroptosis has been demonstrated as an effective strategy to reduce bone loss in inflammatory bone diseases such as osteoarthritis and rheumatoid arthritis (Ge et al., 2022; Behera et al., 2022). Similarly, targeting pyroptosis may alleviate bone destruction in *Staphylococcus* aureus-induced osteomyelitis (Zhu et al., 2019). However, current understanding of pyroptosis in osteomyelitis remains limited, and the regulatory mechanisms involved require further investigation.

### 3.3 Ferroptosis: an emerging iron-dependent RCD in osteomyelitis

Within the iron-overloaded and oxidatively stressed microenvironment of osteomyelitis, ferroptosis occurs as a distinct, iron-dependent form of RCD (Wang et al., 2024). This process is intricately tied to metabolic regulations often governed by HIF-1α, involving the lethal accumulation of lipid peroxides. Its core biochemical features include intracellular iron overload, glutathione (GSH) depletion or inactivation of glutathione peroxidase 4 (GPX4), and the consequent accumulation of lipid peroxides that ultimately lead to plasma membrane rupture (Jiang et al., 2021; Dixon et al., 2012). Analysis of ferroptosis-related gene (FRG) diagnostic models and molecular subtypes significantly associated with immune infiltration provides new insights into the early diagnosis, pathogenesis, and immunotherapy of osteomyelitis (Shi et al., 2023). Key regulators of ferroptosis encompass iron metabolism-related proteins, such as transferrin receptor (TFRC) that mediates iron uptake, and proteins involved in iron storage and release. Lipid metabolism enzymes such as acyl-CoA synthetase long-chain family member 4 (ACSL4) and lysophosphatidylcholine acyltransferase 3 (LPCAT3) are responsible for esterification of polyunsaturated fatty acids, supplying substrates for lipid peroxidation. Antioxidant defense systems include GPX4 (which reduces lipid hydroperoxides to non-toxic lipid alcohols using GSH), the cystine/glutamate antiporter system Xc^−^ (composed of SLC7A11 and SLC3A2, which imports cystine for GSH synthesis), and the newly identified ferroptosis suppressor protein 1 (FSP1, also known as AIFM2) (Wang et al., 2024; Liang et al., 2022; Sun et al., 2023; Chen et al., 2021a; Li W. et al., 2023). Studies on osteomyelitis (OM) have revealed that prostaglandin-endoperoxide synthase 2 (PTGS2, also known as COX-2) is upregulated during *S. aureus* (SA)-induced OM. Silencing PTGS2 or inhibiting its activity suppresses ferroptosis by elevating GPX4 and SLC7A11 protein levels, thereby alleviating inflammation and bone destruction (Zhou SR. et al., 2025). This finding directly links ferroptosis to the pathogenesis of OM. Moreover, the eukaryotic translation initiation factor 5A (EIF5A), known to regulate proliferation, apoptosis, differentiation, and inflammation, is also upregulated in SA-infected bone tissue and promotes ferroptosis and inflammation in osteoblasts during infection (Gao L. et al., 2025). In SA-challenged bone marrow mesenchymal stem cells (BMSCs), increased N6-methyladenosine (m6A) modifications and ferroptosis have been observed. The m6A demethylase FTO was found to suppress SA-induced ferroptosis in BMSCs by modulating the MDM2/TLR4/SLC7A11 signaling pathway. Mechanistically, MDM2 was identified as a downstream target of FTO-mediated m6A demethylation. Upregulation of FTO destabilized MDM2, downregulated TLR4 signaling, and upregulated SLC7A11 and GPX4 in SA-stimulated BMSCs, thereby mitigating ferroptosis (Song M. et al., 2024). Ferroptosis affects nearly all bone-resident cells, including BMSCs, immune cells (Bell et al., 2024), osteocytes, osteoblasts, and osteoclasts (Jiang et al., 2024). In particular, ferroptosis significantly impairs BMSC viability and osteogenic potential within infected bone environments. Targeting ferroptosis in bacterially infected BMSCs has been shown to promote repair of infectious bone defects. For example, Kai Yuan et al. demonstrated that innate immune activation of BMSCs upon bacterial challenge induces phosphorylation and upregulation of interferon regulatory factor 7 (IRF7), which transcriptionally activates ACSL4 and triggers IRF7-dependent ferroptosis. Notably, ferrostatin-1 (Fer-1), a ferroptosis inhibitor, effectively restored osteogenic function in infected BMSCs (Yuan K. et al., 2024). In osteoporotic osteoblasts, epigenetic silencing of GPX4 via DNA methyltransferase (DNMT)-mediated mechanisms was found to promote ferroptosis and contribute to osteoporosis (OP) pathogenesis. Therapeutic strategies targeting DNMTs to preserve GPX4 expression may therefore be beneficial for treating OP and related skeletal disorders (Ruan et al., 2024). Osteoclast overactivity is a hallmark of many bone diseases, and inhibiting osteoclast differentiation through ferroptosis has emerged as a promising strategy (Zhong et al., 2023). Ferroptosis is implicated in osteoclast differentiation during RANKL stimulation, and is driven by iron starvation responses and ferritinophagy. Recent studies have shown that HIF-1α downregulates ferritinophagy and overall autophagic flux under hypoxia. Thus, targeting HIF-1α and ferritin to induce osteoclast ferroptosis may offer a novel therapeutic approach for osteoporosis (Ni et al., 2021), which may also be relevant to osteomyelitis management. In summary, the identification of ferroptosis-related regulators—such as PTGS2, GPX4, and SLC7A11—in SA-induced osteomyelitis positions ferroptosis as a promising and potentially druggable pathway. Its well-characterized molecular underpinnings, involving iron metabolism, lipid peroxidation, and antioxidant defense, offer clear targets for intervention. Preclinical models of OM have demonstrated that modulating these regulators (e.g., PTGS2 inhibition leading to GPX4/SLC7A11 upregulation) can alleviate disease pathology (Zhou SR. et al., 2025), providing a strong theoretical basis for developing or repurposing drugs aimed at specific molecular nodes within the ferroptosis pathway for OM treatment.

### 3.4 Other regulated cell death (RCD) pathways

In addition to the three major RCD pathways discussed above, necroptosis—a regulated form of necrotic cell death mediated by RIPK3-dependent phosphorylation—has emerged as another critical mechanism implicated in inflammatory diseases. This process is primarily driven by the RIPK1-RIPK3-MLKL signaling axis (Ai et al., 2024). In sepsis, stimulator of interferon genes (STING), a central driver of various inflammatory disorders, can be suppressed by inhibiting RIPK3/MLKL signaling, which in turn reduces cell death and dampens STING signaling. This suggests that inhibitors of necroptosis may have extended therapeutic potential in targeting STING and treating sepsis (Zhang X. et al., 2023). The ZBP1-MLKL necroptotic pathway, through its interaction with tumor cell-intrinsic STING signaling, has been shown to drive persistent inflammation and enhance radiation-induced antitumor immunity (Yang et al., 2021). Intra-articular injection of RIPK3 or MLKL inhibitors has been demonstrated to significantly prevent cartilage degeneration and synovial inflammation, revealing potential therapeutic targets for temporomandibular joint osteoarthritis (He et al., 2022). In neurotoxicity-related studies, microglial necroptosis exacerbated neuroinflammation via activation of the JAK2/STAT3 signaling pathway (Yang J. et al., 2025). However, the specific role of necroptosis in osteomyelitis remains to be fully elucidated. PANoptosis is a newly characterized form of inflammatory RCD, orchestrated by a cytosolic multimeric protein complex known as the PANoptosome. This complex integrates multiple RCD pathways—including pyroptosis, apoptosis, and necroptosis—into a highly inflammatory cell death mode (Samir et al., 2020; He et al., 2025). The PANoptosome is typically formed in response to pathogen-associated molecular patterns (PAMPs), damage-associated molecular patterns (DAMPs), or downstream inflammatory cytokines sensed by cytoplasmic pattern recognition receptors (PRRs). One of the key PANoptosome sensors is Z-DNA binding protein 1 (ZBP1) (Karki and Kanneganti, 2023). NLRC5 has been identified as a critical NLR sensor driving PANoptosis and disease pathology. It interacts with NLRP12, NLRP3, and other cell death molecules to form the NLRC5-PANoptosome, which initiates inflammatory cell death. Targeting molecules within this pathway, such as NLRC5 or NLRP12, may prove beneficial in reducing inflammation and improving patient outcomes (Sundaram et al., 2024). NLRP12 has recently been recognized as a key regulator of PANoptosis, with hematopoietic cell kinase (HCK) acting as a modulator of this process. HCK expression is significantly upregulated upon activation of the NLRP12-PANoptosome in response to infection or homeostatic disruption, suggesting its potential as a therapeutic target for inflammation and pathology mitigation (Nadendla et al., 2025). In osteomyelitis, inflammatory cytokines such as IL-1β, IL-6, and TNF-α inhibit osteoblast differentiation and induce apoptosis. Notably, TNF-α-induced suppression of osteoblast differentiation and PANoptosis has been linked to MIR17HG (Li et al., 2024). High-dose TNF-α stimulation can trigger PANoptosis in osteoblasts, where dysregulated inflammatory cell death leads to impaired osteogenic differentiation (Xia et al., 2025). The conceptualization of PANoptosis suggests that cell death during severe infections such as osteomyelitis may not occur through isolated pathways, but rather as a coordinated, synergistic response. Given the highly inflammatory and infectious nature of osteomyelitis, and the pathogen-triggered integration of pyroptosis, apoptosis, and necroptosis within PANoptosis, the formation of PANoptosome-like complexes is likely involved in orchestrating cell death in this disease context. If this hypothesis holds true, targeting the PANoptosome or its individual components may offer greater therapeutic efficacy than inhibiting a single RCD pathway. For example, baicalein has been shown to attenuate multi-organ injury in inflammatory disease models by suppressing mitochondrial damage and mtROS generation, thereby preventing PANoptosome formation and inhibiting PANoptosis (Yuan T. et al., 2024). Interestingly, the ferroptosis inhibitor LPT1 has also been reported to block PANoptosis in models of metabolic-associated fatty liver disease, suggesting that ferroptosis may be integrated into this broader regulatory network (Tong et al., 2023). Collectively, these RCD pathways in osteomyelitis do not operate in isolation. They are triggered and executed within a niche defined by hypoxia and metabolic reprogramming conditions where HIF-1α acts as a central coordinator (Table 2).

**TABLE 2:** Overview of key regulated cell death (RCD) pathways involved in osteomyelitis.

Pathway	Core molecular features/mediators	Major cellular effects in osteomyelitis	References
Apoptosis	Activation of caspase family; formation of apoptotic bodies	Loss of osteoblasts; regulation of immune cell function; resolution (or delay) of inflammation	Josse et al. (2015), Wen et al. (2024), Robinson et al. (2018)
Pyroptosis	Inflammasomes (e.g., NLRP3); Caspase-1/4/5/11; GSDMD; release of IL-1β/IL-18	Intense inflammatory response; cytokine release; enhanced bone resorption and tissue damage	Gao et al. (2025a), Vasudevan et al. (2023), Chen et al. (2024d), Wu et al. (2021)
Ferroptosis	Iron accumulation; lipid peroxidation; dysfunction of GPX4/SLC7A11	Plasma membrane damage; release of inflammatory mediators; impaired function of osteoblasts and BMSCs	Wang et al. (2023b), Zhou et al. (2025a), Chen et al. (2021a), Bell et al. (2024)
Necroptosis	RIPK1–RIPK3–MLKL signaling axis	Pro-inflammatory cell death; tissue injury	Ni et al. (2021), Ai et al. (2024)
PANoptosis	PANoptosome complex (e.g., ZBP1, AIM2, NLRP12); coordinated activation of multiple death pathways	Highly inflammatory and synergistic programmed cell death; potential driver of severe immunopathology	Yang et al. (2025a), Samir et al. (2020), He et al. (2025)

## 4 Regulation of cell death pathways by HIF-1α in osteomyelitis

### 4.1 HIF-1α and apoptosis: protective or detrimental?

The regulatory effect of HIF-1α on apoptosis is highly dependent on cell type and environmental context. In osteoblasts, overexpression of HIF-1α enhances cell activity and promotes osteogenesis (Hannah et al., 2021). Reactive oxygen species (ROS)-mediated activation of caspase-9 increases apoptosis, whereas inhibition of caspase-9 effectively reduces apoptosis. Under oxidative stress, elevated HIF-1α levels can significantly suppress ROS-induced apoptosis (Wang X. et al., 2022). In the field of orthopedics, studies have shown that HIF-1α inhibits osteoblast apoptosis. Knockdown of HIF-1α significantly increases intracellular ROS and apoptosis levels (Xu, 2018), likely through the upregulation of anti-apoptotic factors or modulation of metabolic pathways. Iron overload has been shown to upregulate HIF-1α expression while downregulating RUNX2 and impairing osteogenesis (Zheng J. et al., 2023). Clearance of ROS can enhance PHD2 activity, thereby reducing HIF-1α accumulation, restoring RUNX2 expression, and promoting bone formation (Chen et al., 2024e). If similar protective effects occur in osteoblasts during osteomyelitis, HIF-1α activation may help preserve bone-forming capacity and counteract infection-induced bone loss. Additionally, melatonin has been reported to mitigate LPS-induced osteoblast apoptosis and mitochondrial dysfunction by suppressing the phosphorylation of the mtROS/HIF-1α/PDK1 axis, restoring pyruvate dehydrogenase (PDH) activity, and downregulating lactate production, thereby attenuating metabolic reprogramming and bone damage (Lin Z. et al., 2025). In femoral head osteonecrosis, HIF-1α overexpression promotes BNIP3 expression, which counteracts glucocorticoid-mediated inhibition of hypoxia-induced mitophagy and protects osteocytes from apoptosis (Xu et al., 2021). While HIF-1α promotes osteoclastogenesis under hypoxia via AMPK signaling (Tang et al., 2020), it also induces apoptosis in osteocytes through the JNK/caspase-3 pathway *in vitro*, further stimulating osteoclast differentiation by increasing apoptotic bodies (Song et al., 2020). In immune cells, HIF-1α has been shown to delay neutrophil apoptosis. This may be beneficial during the early stages of infection by extending neutrophil lifespan and enhancing pathogen clearance (Khatib-Massalha et al., 2020; Lu et al., 2021). However, excessive delay in apoptosis can lead to prolonged inflammation and increased tissue damage (Wang et al., 2021; Song Y. et al., 2024). Certain compounds, such as acevaltrate and digitoxin, have been shown to promote apoptosis and inhibit proliferation and migration by suppressing HIF-1α and STAT3 signaling—although these findings were observed in tumor cells (Mi et al., 2022; Mi et al., 2024). Thus, the net effect of HIF-1α on apoptosis—whether protective or detrimental—depends on the specific cell type, disease stage, and the complex interplay within the microenvironment.

### 4.2 HIF-1α and pyroptosis: fueling the fire?

Pyroptosis is a highly inflammatory form of programmed cell death, and its activation in osteomyelitis is closely associated with exacerbated inflammation and bone destruction (Zhu et al., 2019). Emerging evidence suggests a critical regulatory link between HIF-1α and pyroptosis. HIF-1α can directly bind to and activate the promoter region of the NLRP3 gene, thereby increasing the substrate pool for inflammasome assembly. Upon stimulation by bacterial products or ROS, this primed state facilitates Caspase-1 activation and GSDMD cleavage, amplifying pyroptotic responses (Meybodi et al., 2024). Furthermore, HIF-1α can promote pyroptosis through the regulation of inflammatory mediators and via the caspase-8/GSDMD signaling axis (Yang H. et al., 2025), acting in parallel with the classical caspase-1-dependent pathway and aggravating inflammation and tissue damage. In pulpitis—a disease that shares pathological features with osteomyelitis such as bacterial infection, hypoxia, and inflammation—HIF-1α has been shown to activate the NLRP3 inflammasome via the NF-κB signaling pathway, subsequently promoting caspase-1 activation and IL-1β production (Shao et al., 2025). The NLRP3 inflammasome is central to the canonical pyroptosis pathway (Lin M. et al., 2025), and HIF-1α activation under hypoxic conditions can enhance NLRP3 inflammasome activity, leading to pyroptosis. Knockdown of Hif-1α markedly reduces mRNA and protein levels of key pyroptosis-related molecules, including caspase-1, NLRP3, GSDMD, IL-1β, and IL-18. Conversely, HIF-1α activation increases the expression of these molecules, exacerbating inflammatory responses (Zhou L. et al., 2025; Wang Y. et al., 2025). HIF-1α also drives a metabolic shift toward aerobic glycolysis (Warburg effect) in macrophages, upregulating genes such as *PKM2*, *HK2*, and *LDHA*. This shift leads to succinate accumulation in the TCA cycle, which in turn stabilizes HIF-1α in a positive feedback loop, enhances inflammasome activity, and promotes macrophage pyroptosis (Li J. et al., 2025; Aki et al., 2020; Ma et al., 2023). Succinate not only increases intracellular ROS levels but also upregulates IL-1β and TNF-α, further activating NLRP3 inflammasome assembly. For example, Tannahill et al. demonstrated that metabolic dysregulation of the TCA cycle in macrophages activates the succinate–HIF-1α axis, leading to elevated IL-1β and TNF-α levels and increased mitochondrial ROS production, thereby initiating pyroptosis (Tannahill et al., 2013). In renal ischemia-reperfusion injury (RIRI), hypoxia-reoxygenation triggers macrophage infiltration and pyroptosis through the HIF-1α–ROS axis. Here, stabilized HIF-1α upregulates glycolytic enzymes and pro-inflammatory cytokines, while ROS bursts promote NLRP3 inflammasome assembly, leading to GSDMD cleavage and the release of IL-1β and IL-18 (Guo et al., 2024). Notably, salidroside (Sa) alleviates macrophage pyroptosis and inflammation by disrupting the ROS-HIF-1a positive feedback loop. Mechanistically, Salidroside exerts potent antioxidant effects, scavenging excess intracellular ROS. This action restores the activity of prolyl hydroxylases (PHDs), thereby promoting HIF-1α degradation, breaking the positive feedback loop, and ultimately inhibiting the excessive activation of downstream pyroptotic pathways such as the NLRP3 inflammasome (Xueqiang et al., 2025). Similarly, dexamethasone suppresses the HIF-1α–glycolysis axis to reduce airway inflammation and inhibit macrophage pyroptosis (Chen N. et al., 2024). In rheumatoid arthritis (RA) under hypoxic conditions, synovial cell injury involves both BNIP3-mediated mitophagy and NLRP3 inflammasome-mediated pyroptosis. Mitophagy can attenuate hypoxia-induced pyroptosis by clearing ROS and suppressing the HIF-1α/NLRP3 pathway (Hong et al., 2024). HIF-1α also directly promotes the synthesis of pro-inflammatory cytokines such as IL-1β, as shown in osteoarthritic chondrocytes (Zeng et al., 2022). Given that IL-1β is a key effector and product of pyroptosis, this may form a positive feedback loop—HIF-1α → pyroptosis → IL-1β—that sustains the inflammatory process in osteomyelitis. However, in acute ischemia-reperfusion models, overexpression of HIF-1α can reduce pyroptosis markers (e.g., GSDMD cleavage products) and the release of IL-1β and IL-18 by inhibiting the ROS/NLRP3 pathway in microglia. This suggests that HIF-1α may exert dual regulatory roles in pyroptosis depending on the cellular context. Collectively, these findings indicate that HIF-1α generally promotes glycolysis and pro-inflammatory cytokine production, enhancing pyroptosis-related RCD cascades and contributing to bone destruction during infection. Yet under certain injury conditions, it may suppress excessive pyroptosis, reflecting its context-dependent roles in infection-driven inflammation.

### 4.3 HIF-1α and ferroptosis—a critical regulatory axis

#### 4.3.1 Direct molecular links

Emerging evidence strongly suggests that HIF-1α may directly inhibit the occurrence of ferroptosis (Kang et al., 2025). A study on peripheral nerve injury demonstrated that hypoxia suppresses ferroptosis in dorsal root ganglion (DRG) neurons through HIF-1α activation, which upregulates the expression of *SLC7A11* and *GPX4* and increases intracellular cysteine and glutathione levels (An et al., 2024; Qian et al., 2025). Activation of HIF-1α significantly reduces oxidative stress and the expression of ferroptosis markers, an effect that can be reversed by HIF-1α inhibitors (Zhang X. et al., 2025). *SLC7A11* is a key subunit of the cystine/glutamate antiporter system Xc^−^, responsible for importing cystine for GSH synthesis, while *GPX4* is the core enzyme that utilizes GSH to eliminate lipid peroxides and inhibit ferroptosis (Chen et al., 2021b; Tang B. et al., 2022). Enhancing HIF-1α activity markedly reverses the downregulation of *SLC7A11* and *GPX4* and the upregulation of *ACSL4*, while also mitigating the accumulation of MDA, Fe^2+^, and ROS (Yang T. et al., 2025). In osteoarthritis (OA), the HIF-1 signaling pathway has been linked to the transcriptional regulation of opposing ferroptosis mediators. Specifically, HIF-1α correlates with the upregulation of TFRC (which facilitates iron uptake and potentially promotes ferroptosis), while concurrently supporting the expression of SLC7A11 and GPX4 (the core anti-ferroptotic defense system) in chondrocytes and immune cells (Chen W. et al., 2022). HIF-1α can also influence ferroptosis defense systems through metabolic intermediates and signaling pathways. In tumor models, Yang et al. reported that HIF-1α-induced lactate production increases cellular resistance to ferroptosis under acidic conditions, independent of the *SLC7A11* and *FSP1* systems. Additionally, HIF-1α upregulates the expression of the membrane glutamate transporter *SLC1A1*, promoting cysteine uptake and enhancing GSH synthesis—mimicking the *SLC7A11* pathway in resisting ferroptosis (Yang et al., 2023). These findings indicate that HIF-1α may establish an *SLC7A11*-independent ferroptosis-regulating axis via lactate metabolism and alternative amino acid transport pathways. On the other hand, studies on ischemia-reperfusion injury have shown that HIF-1α downregulation lifts the inhibition on *ACSL4*, leading to its significant upregulation and thus promoting ferroptosis and inflammatory responses (Wang Y. et al., 2022). In the context of infectious nonunion, melatonin was found to alleviate osteoblast ferroptosis under infection by modulating the Nox4/ROS/p38 MAPK axis (Qin et al., 2024). Considering the pronounced hypoxic microenvironment within osteomyelitic lesions, HIF-1α is likely activated, while ferroptosis has also been confirmed to participate in the pathogenesis of osteomyelitis. Therefore, HIF-1α likely exerts a suppressive effect on ferroptosis in affected cells by upregulating antioxidant systems, exerting a protective role similar to its anti-apoptotic effects in osteoblasts. Moreover, HIF-1α may regulate the expression of iron metabolism-related genes such as *TFRC*, ferritin, and ferroportin by promoting iron accumulation and metabolic reprogramming, thereby influencing cellular sensitivity to ferroptosis (Karbakhsh Ravari et al., 2025). In OA synovial macrophages, a strong link exists between glycolytic activity and ferroptosis in chondrocytes; inhibiting HIF-1α-mediated glycolysis mitigates chondrocyte ferroptosis and slows OA progression (Li et al., 2025b; Li et al., 2025c). As a transcription factor, HIF-1α can upregulate iron importers such as *TFRC* and *DMT1*, expanding the labile iron pool (Karbakhsh Ravari et al., 2025; Xiong et al., 2022). Propionate was found to dose-dependently suppress HIF-1α expression while upregulating both TFRC and FTH1. Although TFRC facilitates iron uptake, the concurrent upregulation of FTH1 enhances the sequestration and buffering of intracellular iron. This net effect reduces the pool of labile, redox-active iron Fe^2+^, thereby inhibiting ferroptosis and promoting epithelial regeneration despite the increase in iron import markers (Yao et al., 2025). Interestingly, iron ions serve as cofactors for PHD enzymes, which hydroxylate and target HIF-1α for degradation (Lawson et al., 2024; Fiorini and Schofield, 2024; Xing et al., 2025), implying an intrinsic link between HIF-1α and iron metabolism. However, how this interaction specifically affects ferroptosis in the context of osteomyelitis remains to be fully elucidated.

#### 4.3.2 Indirect regulation via inflammatory pathways

HIF-1α can directly bind to the hypoxia response element (HRE) in the promoter region of the pro-inflammatory cytokine IL-6, thereby enhancing the transcription of the IL6 gene (Kou et al., 2023). In rheumatoid arthritis (RA), HIF-1α acts as a transcription factor that directly promotes IL-6 expression by binding to the promoter region of the human IL6 gene, facilitating IL-6 production in B cells (Fan et al., 2023). IL-6 participates in iron metabolism by regulating hepcidin expression through the JAK1/STAT3 pathway. In turn, iron overload promotes the expression of pro-inflammatory cytokines such as IL-6 and IL-1β by disrupting redox homeostasis (Zhang Z. et al., 2022). In degenerative chondrocytes, elevated IL-6 expression triggers lipid peroxidation and iron imbalance via the IL-6/miR-10a-5p/IL-6R axis, thereby promoting ferroptosis and exacerbating inflammation associated with intervertebral disc degeneration (Bin et al., 2021). In inflammatory microenvironments, HIF-1α not only induces IL-6 but also activates the NF-κB pathway to upregulate TNF-α expression (Akhter et al., 2023). In human mast cells (HMC-1), deferoxamine (DFO) treatment upregulates HIF-1α, which subsequently drives the release of pro-inflammatory cytokines such as TNF-α and IL-8 via HIF-1α and NF-κB signaling cascades (Jeong et al., 2003). In Porphyromonas gingivalis–induced periodontitis, HIF-1α exacerbates inflammasome activation in macrophages (Okano et al., 2025). Inflammasomes, by inducing inflammatory cytokine release and suppressing antioxidant defenses, can further amplify ferroptosis. A hallmark of ferroptosis is the lethal accumulation of iron-dependent lipid peroxides within membrane phospholipids. Mechanistically, this process involves three key phases: (Granata et al., 2022): Initiation of lipid peroxidation: Enzymes such as ACSL4 and LPCAT3 facilitate the enrichment of polyunsaturated fatty acids (PUFAs) in cellular membranes, providing the necessary substrates for oxidation; (Rosenberg and Khurana, 2016); Iron-driven amplification: Labile ferrous iron Fe^2+^ acts as a catalyst via Fenton chemistry to convert lipid hydroperoxides into toxic lipid radicals, driving a self-propagating chain reaction of peroxidation; and (Chen H. et al., 2022) Execution due to insufficient GPX4 activity: Ferroptosis occurs when the glutathione (GSH)-dependent enzyme GPX4—which normally reduces lipid hydroperoxides to non-toxic alcohols—is either depleted or overwhelmed by the extent of lipid peroxidation, leading to catastrophic membrane rupture (Wang B. et al., 2023; Tang et al., 2021). HIF-1α directly activates transcription of NADPH oxidase family members such as NOX4 and NOX2, leading to increased intracellular ROS levels under hypoxia (Diebold et al., 2010; Jung et al., 2023). Furthermore, HIF-1α promotes glycolysis while inhibiting mitochondrial oxidative phosphorylation, indirectly destabilizing the electron transport chain and further enhancing ROS accumulation (Cramer et al., 2003). Elevated ROS levels can stimulate NF-κB and inhibit prolyl hydroxylases (PHDs) and factor-inhibiting HIF (FIH), which leads to the cytoplasmic accumulation of HIF-1α and the formation of a pro-inflammatory positive feedback loop (Liang et al., 2021), thereby further accelerating ferroptosis progression.

### 4.4 HIF-1α and other regulated cell death pathways (necroptosis and PANoptosis)

**FIGURE 6:**
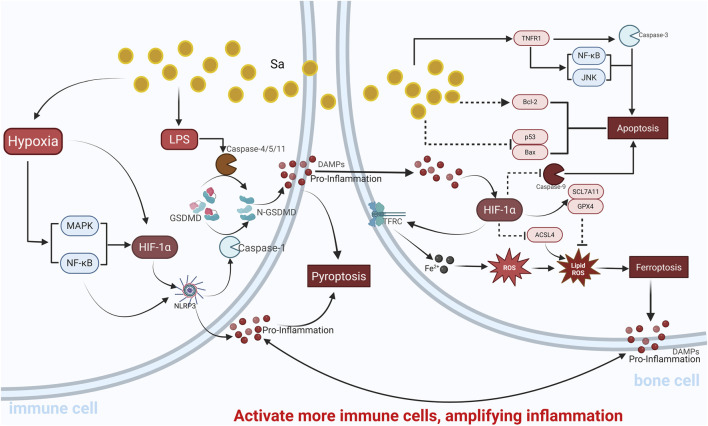
Crosstalk between HIF-1α and regulated cell death pathways in the osteoimmune microenvironment during *Staphylococcus* aureus-induced osteomyelitis. Following *Staphylococcus aureus* (Sa) infection, the osteomyelitic lesion becomes hypoxic. Hypoxia activates HIF-1α through MAPK and NF-κB signaling pathways, leading to downstream inflammasome activation and increased secretion of pro-inflammatory cytokines. Activated caspase-1 cleaves gasdermin D (GSDMD), while Sa-secreted lipopolysaccharide (LPS) directly activates caspase-4/5/11, triggering pyroptosis via N-terminal GSDMD (N-GSDMD) pore formation. In turn, immune cells release pro-inflammatory factors that enhance HIF-1α activation in osteoblasts, increasing the expression of transferrin receptor (TFRC) and causing intracellular iron overload, thereby promoting ferroptosis and amplifying inflammation. HIF-1α also modulates ferroptosis by upregulating SLC7A11 and GPX4, and downregulating ACSL4. In parallel, HIF-1α suppresses caspase-9 activity, thereby attenuating apoptosis. Sa infection also promotes apoptosis through multiple mechanisms: its surface protein A (SpA) upregulates pro-apoptotic factors p53 and Bax, and downregulates the anti-apoptotic protein Bcl-2. Additionally, SpA can engage TNFR1, activating the NF-κB and JNK pathways and ultimately leading to caspase-3-dependent apoptosis. These regulated cell death processes (pyroptosis, ferroptosis, and apoptosis) collectively contribute to the release of damage-associated molecular patterns (DAMPs), which recruit and activate additional immune cells, further exacerbating inflammation.

In arthritis, RIPK3-induced necroptosis in intestinal epithelial cells exacerbates disease progression. HIF-1α, as a critical transcriptional repressor, can directly downregulate RIPK3 expression, thereby limiting necroptosis. Deletion of HIF-1α significantly enhances RIPK3-mediated necroptosis, leading to aggravated inflammation (Lyu et al., 2024). In inflammatory macrophages, HIF-1α upregulates specific microRNAs and promotes necroptosis through ATP depletion. Studies have shown that HIF-1α activation enhances RIP1/RIP3/MLKL signaling in macrophages via miR-210 and miR-383, aggravating necroptosis in ischemic brain injury models. Similar mechanisms have been observed in atherosclerotic macrophages, where HIF-1α facilitates energy depletion and necroptosis through microRNA pathways (Karshovska et al., 2020; Luo et al., 2022). In atherosclerosis, C-type natriuretic peptide (CNP) promotes anti-inflammatory macrophage polarization and efferocytosis, while reducing foam cell formation and necroptosis by enhancing the interaction between PHD2 and HIF-1α, thus accelerating HIF-1α degradation (Bao et al., 2024). In ischemic brain injury models, oxygen-glucose deprivation (OGD) and middle cerebral artery occlusion (MCAO) induce cell death, regional cerebral ischemia, and neurological deficits primarily through HIF-1α upregulation-mediated necroptosis (Yang et al., 2017). PANoptosis is a unique form of regulated cell death mediated by the interaction of multiple complexes, including the PYRIN inflammasome, apoptosome, and necrosome. In the context of osteogenic differentiation and inflammatory microenvironments, miR-18a has been identified as a negative regulator of the HIF-1α/NLRP3 axis, thereby suppressing PANoptosis and influencing osteogenic differentiation. Specifically, miR-18a downregulates HIF-1α expression and reduces NLRP3 activation, attenuating the mixed RCD characteristics of PANoptosis (Zhang et al., 2024a). Oxidative stress promotes the progression of PANoptosis. HIF-1α serves as both a sensor and an effector in this process: on the one hand, hypoxia and ROS signals activate HIF-1α transcriptional activity; on the other hand, HIF-1α upregulates several PANoptosis-related genes (e.g., NLRP3, RIPK3), establishing a positive feedback loop that drives widespread activation of cell death pathways (Gao et al., 2024). In summary, HIF-1α not only modulates necroptosis through transcriptional repression and microRNA-mediated mechanisms but also coordinates and transitions between different RCD modalities via regulation of the NLRP3 inflammasome and PANoptotic complexes (Figure 6).

## 5 Emerging therapies: HIF-1α and cell death modulation in osteomyelitis

### 5.1 Targeting HIF-1α: a double-edged sword?

The osteomyelitis microenvironment is often characterized by localized hypoxia, which induces the upregulation of HIF-1α and drives various pathological responses, including inflammation and pro-inflammatory forms of cell death such as pyroptosis. Inhibiting HIF-1α activity may offer therapeutic potential for controlling osteomyelitis. Notably, studies have shown that HIF-1α inhibitors can alleviate inflammation and enhance osteogenic capacity in mouse models of *S. aureus*-induced osteomyelitis (Zhang et al., 2022a). On the other hand, HIF-1α is a crucial regulator of bone regeneration and angiogenesis. Its activation promotes osteocyte survival under hypoxic conditions and enhances bone repair (Song S. et al., 2023; You et al., 2023; Han et al., 2024). Moreover, HIF-1α also modulates immune cell function, enhancing the bactericidal activity of macrophages and other immune cells. Therefore, excessive inhibition of HIF-1α might impair the host’s ability to eliminate infection (Bhandari and Nizet, 2014). For example, treatment with IDF-11774, a HIF-1α inhibitor, significantly suppressed bone formation defects in *S. aureus*-infected models. Serum levels of IL-6, IL-1β, and CRP were markedly reduced in infected mice after IDF-11774 administration, indicating that HIF-1α inhibition contributes to the attenuation of infectious inflammation (Zhang et al., 2022a). While HIF-1α suppression can mitigate bone destruction and inflammation, care must be taken to avoid compromising bone remodeling and immune defense. Moderate HIF-1α inhibition in the early stages of infection may improve bone healing, whereas relaxation of inhibition strategies may be necessary during the bone repair phase. Other known HIF-1α inhibitors include cardiac glycosides (e.g., digoxin) and estrogen derivatives such as 2-methoxyestradiol, though evidence for their application in osteomyelitis is currently lacking. Systemic HIF-1α inhibition may impair its beneficial effects or even exacerbate tissue injury. This suggests that moderate activation of HIF-1α during bone defect repair may promote osteoangiogenic regeneration. In intervertebral disc degeneration (IVDD), HIF-1α maintains mitochondrial integrity and glycolysis through PDK-1, thereby protecting nucleus pulposus cells from excessive oxidative stress, highlighting its potential in antioxidative stress responses (Liu et al., 2024). Prolyl hydroxylases (PHDs) hydroxylate HIF-1α at Pro402 and Pro564 within the oxygen-dependent degradation domain (ODD), targeting it for degradation (Zhang J. et al., 2025). Hypoxia mimetics and PHD inhibitors (e.g., DMOG) can stabilize HIF-1α and mimic hypoxic signaling. In tissue engineering studies, DMOG-loaded biomaterials have been shown to significantly enhance osteogenic differentiation of mesenchymal stem cells and angiogenesis of endothelial cells (Han et al., 2024). Clinically, PHD inhibitors such as roxadustat have been approved for anemia treatment. However, roxadustat may exacerbate inflammatory infections due to its immunosuppressive potential, and unintended side effects must be considered (Zhu et al., 2022). Iron chelators such as deferoxamine (DFO) can indirectly stabilize HIF-1α by reducing intracellular Fe^2+^ levels, and have shown various effects including stem cell regulation, immune modulation, vascular remodeling, and osteogenesis (Zhu et al., 2023). In bone defect interventions, sustained DFO release to activate HIF-1α signaling has been shown to regulate the expression of genes associated with osteoblast differentiation and bone mineralization in BMSCs, thereby promoting osteogenesis and vascularized regeneration (Shan et al., 2025). It may also alleviate inflammation by increasing HIF-1α expression (Zhang et al., 2022c). Nevertheless, in the context of bone infection or defects, while stabilizing HIF-1α may enhance bone regeneration, it might also promote bacterial tolerance and dissemination (Holden et al., 2016). Thus, activation strategies should be combined with antimicrobial therapy. Overall, HIF-1α contributes both to pathological progression and to bone healing and immune function in osteomyelitis, representing a dual-role factor. Its modulation—either inhibition or activation—may offer both benefits and risks. The net therapeutic effect will largely depend on disease stage, dominant pathological processes, and the ability to selectively regulate HIF-1α activity. Future research should further clarify the cell-specific and stage-specific functions of HIF-1α in osteomyelitis to enable precise therapeutic targeting.

### 5.2 Regulation of specific RCD pathways

#### 5.2.1 Inhibition of ferroptosis

Emerging evidence suggests that ferroptosis-associated oxidative stress pathways may contribute to the pathogenesis of osteomyelitis. While definitive mechanistic evidence remains limited, the observation of iron overload and lipid peroxidation markers in osteomyelitis models indicates that these pathways likely promote cellular damage and inflammation (Gao L. et al., 2025). Thus, inhibiting ferroptosis holds therapeutic promise for ameliorating osteomyelitis-related pathology. The PTGS2 inhibitor Etoricoxib has demonstrated therapeutic effects in murine models of osteomyelitis, modulating ferroptosis-associated oxidative stress pathways (Zhou SR. et al., 2025). With growing insights into the mechanisms of ferroptosis, several proteins have been identified that defend against this process by limiting lipid peroxidation, including glutathione peroxidase 4 (GPX4), ferroptosis suppressor protein 1 (FSP1), and GTP cyclohydrolase 1 (GCH1) (Bersuker et al., 2019; Kraft et al., 2020). GPX4 utilizes reducing equivalents from glutathione (GSH) to specifically reduce toxic phospholipid hydroperoxides within cellular membranes to non-toxic lipid alcohols, thereby preventing lethal membrane damage and promoting cell survival (Zhang et al., 2024b). In diabetic nephropathy models, both nanomaterials and pharmacological agents that upregulate GPX4 expression have been shown to alleviate ferroptosis-induced damage to varying degrees (Liu et al., 2025; Wu et al., 2025). In *S. aureus*-induced osteomyelitis, SA suppresses GPX4 expression to promote ferroptosis. Notably, recent studies report that inhibiting death-associated protein kinase 3 (DAPK3) markedly upregulates GPX4 and alleviates osteomyelitis progression (Kou et al., 2025). GSH, a critical cofactor for GPX4’s lipid peroxide detoxification, is synthesized via the cystine/glutamate antiporter System Xc^−^, primarily regulated by SLC7A11 (Chen H. et al., 2023). In lung cancer studies, downregulation of SLC7A11 has been employed to disrupt the SLC7A11/GPX4 axis, impairing antioxidant defenses and enhancing ferroptosis sensitivity (Shi et al., 2025). Upregulation of SLC7A11 enhances GSH synthesis and suppresses ferroptotic signaling, making it a compelling target for therapeutic intervention (Zhao Y. et al., 2025).

Beyond the classical System Xc^−^/GPX4 pathway, cells possess alternative, GPX4-independent antioxidant defense systems to resist ferroptosis. The FSP1-CoQ10-NAD(P)H axis represents one such major mechanism. FSP1 functions independently of GSH, reducing coenzyme Q10 (CoQ10) to CoQ10H2 via NADPH, which quenches lipid peroxyl radicals and inhibits ferroptosis even in the absence of GPX4 (Lv et al., 2023; Zheng X. et al., 2023; Doll et al., 2019). While the role of FSP1 has been explored in chondrocyte ferroptosis (Wang S. et al., 2022), most research currently focuses on cancer. Similarly, the GCH1-BH4 axis constitutes another autonomous regulatory system. As the rate-limiting enzyme for tetrahydrobiopterin (BH4) synthesis, GCH1 converts GTP to BH4, a potent lipid antioxidant capable of directly scavenging lipid peroxides (Kraft et al., 2020; Zhou et al., 2024). GCH1 has been shown to inhibit LPS-induced ferroptosis in macrophages (Xiao et al., 2023) and attenuate oxidative stress in spinal cord injury models (Chen et al., 2024g), suggesting its potential relevance in the inflammatory milieu of osteomyelitis. In atherosclerosis, GCH1 activation reduces mitochondrial oxidative stress and inhibits endothelial ferroptosis, thereby limiting disease progression (Du et al., 2024).

ACSL4 promotes ferroptosis by facilitating the esterification of polyunsaturated fatty acids (PUFAs) into acyl-CoAs. Hence, targeting ACSL4 is also a promising strategy for ferroptosis-related diseases (Huang et al., 2024). In inflammatory bowel disease, fibroblast-overexpressed ACSL4 reprograms lipid metabolism and sensitizes intestinal epithelial cells to ferroptosis, exacerbating colitis. Pharmacological inhibition or genetic deletion of fibroblast ACSL4 alleviates colitis severity (Huang et al., 2025). Rociletinib, a potent ferroptosis inhibitor, covalently binds to Cys170 of ACSL4 to suppress its enzymatic activity, thereby reducing lipid peroxidation and ferroptosis, and mitigating ferroptosis-induced liver damage in acute liver injury (Linghu et al., 2025). Although PTGS2 inhibition currently represents a promising therapeutic approach to combat ferroptosis in osteomyelitis, the fundamental mechanisms of ferroptosis—iron dysregulation, lipid peroxidation, and GPX4/SLC7A11 dysfunction—suggest a broader array of potential therapeutic targets. Many such ferroptosis regulators, including ferrostatin-1 and iron chelators, have shown efficacy in other ferroptosis-related diseases. Applying these direct modulators in osteomyelitis models represents an important future direction for therapeutic development.

#### 5.2.2 Inhibition of pyroptosis

Pyroptosis is a key driver of inflammation and bone destruction in osteomyelitis (Gao L. et al., 2025). Activation of caspase-1 leads to the cleavage and release of interleukin-1β (IL-1β), resulting in pyroptotic cell death (Güneş et al., 2025). Inhibiting pyroptosis-related proteins—such as caspase-1 inhibitors and gasdermin D (GSDMD) blockers—has been shown to attenuate *S. aureus*-induced pyroptosis and bone damage both *in vitro* and *in vivo* (Zhu et al., 2019; Song M. et al., 2023). Suppression of pyroptosis can markedly reduce oxidative stress and inflammatory responses while improving cellular viability (Yang X. et al., 2025). In diabetic foot ulcers (DFUs), intracellular accumulation of *S. aureus* has been shown to induce pyroptosis in keratinocytes, contributing to persistent and recurrent bacterial colonization and chronic inflammation. Targeting the pyroptosis pathway may help eliminate intracellular bacterial niches, promote inflammation resolution, and facilitate wound healing (Pastar et al., 2021). In spinal cord injury, inhibiting pyroptosis in microglia via the PI3K/AKT/NF-κB and NLRP3/caspase-1/GSDMD signaling axes has been found to significantly enhance axonal regeneration and motor function recovery (Gao J. et al., 2025). In acute gouty arthritis, traditional Chinese medicine formulations have been used to suppress caspase-1 activation and IL-1β production, thereby inhibiting NLRP3 inflammasome activation and pyroptosis in THP-1 cells, ultimately improving disease outcomes (Wang N. et al., 2025). In sepsis-induced acute lung injury, uncontrolled inflammatory responses driven by pyroptosis of alveolar macrophages are a major pathological feature. Hong Li and colleagues demonstrated that treatment with POPAA-1 effectively suppressed macrophage pyroptosis via inhibition of the NF-κB and NLRP3/caspase-1/GSDMD pathways, thereby alleviating lung injury. Pinocembrin (PIN), a flavonoid compound extracted from *Pinus sylvestris*, selectively suppresses LPS- and RANKL-induced IL-1β release, effectively inhibiting both pyroptosis and osteoclastogenesis (Zhang W. et al., 2025). Its potent anti-inflammatory properties warrant further investigation in osteomyelitis. In addition to herbal and biological agents, trace element regulation also plays a role in pyroptosis. Zinc deficiency is closely linked to oxidative stress, inflammation, and programmed cell death (Zhang Q. et al., 2022). Zinc deficiency upregulates key pyroptosis mediators, while zinc supplementation can mitigate oxidative damage and suppress the expression of pyroptosis-associated pathways and factors (Cai Z. et al., 2025). Overall, inhibiting pyroptosis within the inflammatory microenvironment of osteomyelitis has emerged as a promising strategy to halt disease progression and improve clinical outcomes.

#### 5.2.3 Regulation of apoptosis

In osteomyelitis, increased apoptosis of osteoblasts and other bone-associated cells is a key mechanism contributing to impaired bone formation and bone loss (Marriott, 2013). Broad-spectrum or specific inhibition of caspase activity—for example, the use of 50 μM Z-VAD-FMK in a *Brucella abortus* infection model—can significantly reduce the Annexin V-positive rate of osteoprogenitor cells, suppress apoptosis in osteoblasts and macrophages induced by infection, and alleviate bone and joint destruction (Scian et al., 2012). In co-culture systems of *S. aureus* with human or murine osteoblasts, pan-caspase inhibition reduces nuclear fragmentation and DNA laddering, indicating its suppressive effect on infection-induced apoptosis (Ning et al., 2011b). Following *S. aureus* infection, osteoblasts specifically upregulate TRAIL receptors DR4/DR5 while downregulating the secreted decoy receptor OPG, increasing cellular sensitivity to TRAIL and initiating caspase-8-mediated apoptosis (Young et al., 2011). Application of neutralizing anti-TRAIL antibodies can dose-dependently inhibit caspase-8 activity and reduce infection-induced apoptosis of osteoblasts, suggesting that blockade of TRAIL signaling is a viable therapeutic strategy (Alexander et al., 2003). Allicin, an active compound derived from garlic, reverses steroid-induced osteoblast apoptosis by activating the PI3K/Akt signaling pathway, and alleviates bone necrosis in in vivo models, highlighting the anti-apoptotic potential of this pathway (Zhan et al., 2020). Dexamethasone (Dex)-induced osteoblast apoptosis involves the ROS–PI3K/Akt/GSK3β pathway. Activation of PI3K/Akt or knockdown of GSK3β can upregulate Bcl-2 and inhibit the cleavage of caspase-3/9, thereby significantly reducing the rate of apoptosis (Deng et al., 2019). Bcl-2, a key anti-apoptotic molecule in the mitochondrial pathway, prevents cytochrome c release and inhibits downstream caspase-3 activation. Bcl-2−/− mice exhibit dysfunction in both osteoblasts and osteoclasts, whereas restoration of Bcl-2 significantly reduces TUNEL-positive osteoblasts and improves bone mass (Nagase et al., 2009). Balancing the expression of Bcl-2 and pro-apoptotic members such as Bim and Bad can precisely regulate cell fate, offering a strategy to protect healthy bone cells during osteomyelitis. Inducing apoptosis in persistently infected cells or overly activated inflammatory immune cells may facilitate clearance of infectious foci and control inflammation. Infected cells undergoing programmed apoptosis can enclose bacteria within apoptotic bodies, which are subsequently eliminated by efferocytosis through phagocytes, thereby reducing bacterial dissemination and long-term persistence (Vu et al., 2024). Moreover, apoptosis of hyperactivated immune cells drastically decreases pro-inflammatory cytokine release, helping to prevent sustained inflammation and strategic tissue repair failure (Kilinç et al., 2021). Appropriately inducing apoptosis in infected or overly activated immune cells to eliminate refractory infection reservoirs and interrupt chronic inflammatory cycles may provide an innovative host-targeted therapeutic strategy for chronic bone infections such as osteomyelitis (Table 3).

**TABLE 3:** Emerging therapeutic strategies targeting HIF-1α and regulated cell death in osteomyelitis.

Therapeutic target	Strategy/example drug	Proposed mechanism in osteomyelitis	References
HIF-1α Axis	HIF-1α inhibition (IDF-11774)	Suppresses HIF-1α–driven inflammation during the inflammatory phase, improves osteogenic function	Zhang et al. (2022a)
	PHD inhibition (Roxadustat)	Stabilizes HIF-1α during the repair phase, promoting angiogenesis and tissue regeneration	Han et al. (2024), Zhu et al. (2022)
Ferroptosis Pathway	PTGS2 inhibition (Etoricoxib)	Inhibits PTGS2, upregulates GPX4/SLC7A11, reduces ferroptosis, and alleviates inflammation	Zhou et al. (2025a)
	GPX4/SLC7A11 activation (Ferrostatin-1)	Directly blocks lipid peroxidation, protecting cells from ferroptotic death	Zhao et al. (2025a), Zhang et al. (2024c)
Pyroptosis Pathway	Caspase-1 inhibition (Ac-YVAD-cmk)	Inhibits Caspase-1 activity, reduces IL-1β release and pyroptosis	Pastar et al. (2021), Gao et al. (2025b), Wang et al. (2025b)
	GSDMD inhibition	Prevents GSDMD pore formation, blocking the execution phase of pyroptosis	Coll et al. (2022)
PANoptosome	Inhibition of ZBP1/core components	Shuts down synergistic inflammatory cell death pathways, fundamentally controlling immune pathology	Karki and Kanneganti (2023)
Combination/Multimodal	Drug-loaded nanoscaffolds (antibiotics + RCD inhibitors + growth factors)	Enables localized, sustained, and multi-target modulation of pathological processes while promoting repair	Yuan et al. (2024a), Liu et al. (2022b), Yekani et al. (2024)

## 6 Discussion and future perspectives

Recent studies have revealed that within the hypoxic immune microenvironment of osteomyelitis, hypoxia-inducible factor-1α (HIF-1α) serves as a central transcription factor, mediating metabolic reprogramming and regulating various forms of regulated cell death (RCD). Under hypoxic conditions, HIF-1α accumulates stably and promotes macrophage polarization toward a glycolytic, pro-inflammatory M1 phenotype, enhancing the secretion of inflammatory cytokines such as TNF-α and IL-1β, thereby exacerbating bone tissue inflammation (Li J. et al., 2025). Taken together, HIF-1α functions as a “metabolism-inflammation” hub, linking hypoxic stress, ROS generation, and multiple RCD pathways. Metabolic stress enhances HIF-1α activity, triggering downstream signaling cascades that create a vicious cycle of inflammation and tissue damage.

In the context of pyroptosis, existing evidence confirms that HIF-1α plays a dual regulatory role. On one hand, HIF-1α upregulates the transcription and expression of the NLRP3 inflammasome, activates caspase-1, and induces gasdermin D (GSDMD)-mediated pyroptosis, leading to the release of pro-inflammatory cytokines such as IL-1β and IL-18 (Zhao P. et al., 2025). On the other hand, HIF-1α can interestingly upregulate BNIP3-mediated mitophagy (an apoptosis-like autophagy), which eliminates damaged mitochondria and reduces mitochondrial ROS, thereby mitigating excessive pyroptotic responses (Hong et al., 2024). Thus, HIF-1α both initiates and regulates pyroptosis, forming a dynamic feedback network in the osteomyelitis microenvironment. Conversely, the release of inflammatory cytokines (e.g., IL-1β/IL-18) during pyroptosis amplifies local inflammation. Elevated expression of pyroptosis markers in osteomyelitis patients and models has been correlated with osteoclast activity and bone resorption, suggesting that pyroptosis may indirectly accelerate bone destruction by intensifying inflammation.

Regarding ferroptosis, a critical analysis of the literature points to an apparent discrepancy in the regulatory relationship between HIF-1α and ferroptosis. This review highlights a clear dualism: HIF-1α can exert a cytoprotective function by directly upregulating anti-ferroptotic defenses like SLC7A11 and GPX4. However, Chen et al. reported that under inflammatory conditions, HIF-1α upregulates the transferrin receptor (TFRC), promoting iron uptake and Fenton reaction activation, which results in chondrocyte ferroptosis (Chen BY. et al., 2024). We propose that this paradox can be resolved by considering the dominant “net effect” within the specific pathological context of osteomyelitis. We hypothesize that in the highly inflamed and hypoxic microenvironment, the pro-pathological, indirect effects of HIF-1α (i.e., inflammation, ROS, and TFRC-mediated iron accumulation) dramatically overwhelm the weaker, direct cytoprotective signaling. Consequently, the overall contribution (net effect) of HIF-1α stabilization in osteomyelitis is to promote ferroptosis and accelerate tissue destruction. This model explains why inhibiting HIF-1α (e.g., with IDF-11774) leads to a net inhibition of ferroptosis by shutting down its dominant, pro-pathological arm.

Beyond individual pathways, the simultaneous occurrence of RCD forms points toward a more complex phenomenon: PANoptosis. Within osteomyelitis lesions, mitochondrial damage and metabolic stress can trigger multiple death signals simultaneously. For instance, ferritin released during pyroptosis can serve as a substrate for ferroptosis, while autophagy (e.g., ferritinophagy) mediates crosstalk between the two (Zhao P. et al., 2025; Sun et al., 2022). Against this background, the concept of PANoptosis—defined as the simultaneous activation of pyroptosis, apoptosis, and necroptosis—has gained attention. Chang-Liang Xia et al. reported that TNF-α can induce concurrent pyroptosis, apoptosis, and necroptosis in osteoprogenitor cells (Xia et al., 2025). Furthermore, Zhang et al. found that miR-18a downregulates the HIF-1α/NLRP3 pathway, thereby inhibiting TNF-α-induced PANoptosis (Zhang et al., 2024a). Based on this evidence, we propose the “Metabolic Stress–HIF-1α–PANoptosome Axis” model. This posits that under metabolic stress, a HIF-1α activation complex coordinates multiple death pathways simultaneously. We also propose a “HIF-1α–IL-1β–ACSL4 Positive Feedback Loop,” where inflammatory signals enhance HIF-1α activity and upregulate ferroptosis effectors such as ACSL4, amplifying cell damage (Wang Y. et al., 2022).

From a therapeutic perspective, multi-target modulation of the HIF-1α-RCD axis is particularly promising. Studies have shown that the HIF-1α inhibitor IDF-11774 restores osteogenic marker expression and reduces serum levels of IL-6 and IL-1β in *S. aureus*-induced osteomyelitis models (Cao et al., 2024). Based on this, combinatorial strategies could be devised, such as combining HIF-1α inhibitors with ferroptosis inducers or pyroptosis inhibitors. Additionally, nanocarriers or biodegradable biomaterials (e.g., dual-functional hydrogels) targeting HIF-1α could be developed to improve specificity and enhance regenerative outcomes (Shi et al., 2024). Other compounds, such as the NLRP3 inhibitor MCC950 and monomeric glycosides (MDP), have also shown potential in disrupting the ROS–HIF-1α–NLRP3 cascade (Hong et al., 2022).

Future research should focus on several key areas: (Granata et al., 2022): Cell-Type Specific Functions–Elucidating the distinct roles of HIF-1α in macrophages, osteoblasts, and osteoclasts; (Rosenberg and Khurana, 2016); Upstream Regulation and Downstream Effectors–Exploring how epigenetic factors and miRNAs regulate HIF-1α, and its influence on inflammasomes and GPX4; (Chen H. et al., 2022); Metabolic Crosstalk–Investigating the interaction between HIF-1α and other metabolic signals (HIF-2α, SIRT, SDH); (Masters et al., 2022); Translational Therapeutics–Evaluating precision intervention strategies.

In summary, HIF-1α lies at the intersection of the bone–immune microenvironment in osteomyelitis. Its role in RCD regulation opens new avenues for therapeutic targeting and warrants further validation through *in vitro* and *in vivo* studies. Future efforts should focus on elucidating the specific functions of different HIF-1α isoforms across cell types and unraveling the intricate crosstalk among various RCD pathways, especially to confirm the central role of PANoptosis in osteomyelitis. Therapeutic strategies should move toward precision and personalization—shifting from broad-spectrum antibiotics and HIF-1α inhibitors to stage-specific, cell-targeted interventions. Ultimately, smart nanodelivery systems capable of co-delivering antibiotics, RCD inhibitors, and regenerative factors hold promise for simultaneously addressing the three major pathological components of osteomyelitis: infection, inflammation, and bone loss. As our understanding of this central regulatory axis deepens, we foresee a paradigm shift beyond conventional anti-infective therapies—toward host-directed interventions that modulate immunity and cell death responses, paving the way for transformative breakthroughs in the treatment of osteomyelitis.

## 7 Conclusion

The intricate interplay between HIF-1α and various forms of regulated cell death (RCD) represents a central axis in the pathophysiology of osteomyelitis. A deeper understanding of this regulatory network not only offers novel insights into the mechanisms underlying disease progression but also opens up promising avenues for developing innovative therapeutic strategies against this refractory orthopedic infection. Translational success will depend on the ability to design interventions that are both cell type–specific and temporally targeted, aiming to harness the protective effects of RCD modulation while minimizing detrimental outcomes. As research continues to unravel the roles of emerging RCD pathways—particularly ferroptosis—in osteomyelitis, we are increasingly equipped with the conceptual and technological tools needed to intervene more effectively and improve patient outcomes.
